# A multi-objective fuzzy programming model for port tugboat scheduling based on the Stackelberg game

**DOI:** 10.1038/s41598-024-76898-6

**Published:** 2024-10-23

**Authors:** Yangjun Ren, Qiong Chen, Yui-yip Lau, Maxim A. Dulebenets, Botang Li, Mengchi Li

**Affiliations:** 1 School of Economics and Trade, Changzhou Vocational Institute of Textile and Garment, Changzhou, 213164 China; 2 Navigation College, Jimei University, Xiamen, 361021 China; 3 Division of Business and Hospitality Management, College of Professional and Continuing Education, The Hong Kong Polytechnic University, Hong Kong, China; 4 Department of Civil and Environmental Engineering, Florida A&M University-Florida State University, Tallahassee, FL 32310 USA; 5 Department of Port and Shipping Management, Guangzhou Maritime University, Guangzhou, 510725 China; 6 School of Shipping Economics and Trade, Guangzhou Maritime University, Guangzhou, 510725 China

**Keywords:** Tugboat scheduling, Stackelberg game, Fairness principle, Task random arrival, Fuzzy programming, Environmental sciences, Environmental social sciences, Mathematics and computing

## Abstract

To solve the optimization problem of tugboat scheduling for assisting ships in entering and exiting ports in uncertain environments, this study investigates the impact of the decisions of tugboat operators and port dispatchers on tugboat scheduling under the scenario of dynamic task arrival and fuzzy tugboat operation time. Considering the features of the shortest distance tugboat principle, the first available tugboat principle, and the principle of fairness in the task volume of each tugboat, the tugboat company aims to minimize the total daily fuel consumption of tugboat operations, maximize the total buffer time of dynamic tasks, and minimize the total completion time as the objective functions. Due to the limitations of port vessel berthing and departure, as well as the allocation standards for piloting or relocating tugboats, the present study proposes a Stackelberg game-based fuzzy model for port tugboat scheduling with the tugboat operator and port dispatcher acting as decision makers at the upper and lower levels, respectively. A seagull optimization algorithm based on priority encoding and genetic operators is designed as a solution approach. CPLEX, genetic algorithm, standard seagull optimization algorithm, and simulated annealing algorithm are used to compare and analyze the solution results for the 45 problem cases generated from the actual data obtained from the Guangzhou Port. The results verify the efficiency of the proposed seagull optimization algorithm based on priority encoding and genetic operators. Furthermore, additional experiments are conducted to evaluate the changes in fairness coefficient, uncertain parameter correlation coefficients, and objective function correlation coefficients to demonstrate the practicality of the fuzzy programming model. This analysis involves adjusting the confidence level incrementally from 0 to 100% with respect to the model’s uncertain parameters.

**Supplementary Information:**

The online version contains supplementary material available at 10.1038/s41598-024-76898-6.

## Introduction

The overall cargo throughput of Chinese ports still maintains rapid growth. In 2022, the total cargo throughput of ports nationwide reached 15.68 billion tons, which is a year-by-year increase of 0.9%. Against the backdrop of slowing economic growth and accelerating industrial restructuring, China’s port throughput still maintains a steady growth trend^1^. The Shanghai International Shipping Center has been built, and the maritime liner connectivity index is firmly ranked first in the world. According to the recent statistics, maritime transportation accounts for over 95% of China’s foreign trade cargo transportation volume^2^.

In trade transactions, ports, as an important component of modern logistics chains, are the starting and ending points of maritime transportation and are also a comprehensive logistics system that should provide safe entry, exit, and berthing for vessels, including green port investments^3^, various buildings, facilities, machinery, tools, and personnel^4^. The operations at port waters (referring to waters surrounding berths, waterways, and anchorages) cannot be separated from tugboats. As a tool, tugboats assist ships entering and leaving the port by providing necessary berthing, shifting, and piloting services. They are an important port resource for achieving port functions. However, the availability of tugboats and the ability of each tugboat to provide services to the arriving vessels are limited. Therefore, in order to improve the efficiency of tugboat operations, tasks are expected to be dispatched through scientific methods and analytical tools. Tugboat scheduling is an important issue that urgently needs to be addressed to improve the efficiency and competitiveness of port operations.

In the process of tugboat operations, objective entities, such as vessels, shipping agents, port dispatchers, pilot companies, barge operators, pilots, tugboats, etc., are involved, among which the port dispatchers and tugboat companies are the main entities in formulating tugboat dispatch plans for port operations^5^. At present, in the actual planning operation of tugboat scheduling at the Guangdong Province ports, the port scheduling party processes large vessels into each independent operation based on customer requests and determines the tugboat power and quantity matching plan required for each operation according to the “Guangdong Province Port Ship Berthing, Departure, Pilot or Transfer Tug Ship Allocation Standard”^6^, and then the key information is entered into the scheduling management system. The tugboat operator prepares a specific tugboat operation plan based on this information and directs the tugboat deployment. The initial matching plan formulated by the port scheduling party needs to be adjusted based on the number of tugs owned by the tugboat operator and the actual operational conditions of a given day. As such, there are direct interactions between the tugboat operator and the port scheduling party.

Under the guidance of the tugboat and task matching scheme provided by the port dispatching party, the tugboat operator often uses the shortest distance tugboat principle, the first available tugboat principle, and the principle of fairness in the task quantity of each tugboat to assign tugboats. Among them, the principle of shortest distance tugboat operation (TSD) refers to first selecting the tugboat with the shortest distance (referring to the distance between the tugboat’s location after completing the previous task and the current task location or the distance between the tugboat’s berthing base and the current task location) as the scheduling plan according to the matching rules between the tugboat and the vessel and then dispatching that tugboat to provide services to the vessels to be served. The First Available Tug Principle (FAT) refers to first selection of tug schemes that meet the requirements of the vessel based on the matching rules between tugs and vessels. From the available tug schemes, the tug scheme that completes the previous operation as quickly as possible and is idle is selected as the adopted scheme, allowing it to provide services for the vessel. The principle of shortest distance tugboat operation is very similar to the principle of the first available tugboat in some cases, with the difference being that the focus of the TSD principle is on how to quickly operate for the next vessel, while the FAT principle considers the overall management of all tugboat operations. The principle of fairness in the task volume of each tugboat (UWAT) is to ensure that the same tugboat driver does not experience excessive fatigue in the same shift and ensures sufficient rest time. Many tugboat operators provide compensation to tugboat drivers based on the number and importance of tasks they participate in. Therefore, to ensure fairness in the salaries of tugboat drivers, tugboat dispatchers allocate tugboat tasks evenly over time, ensuring that the number of tasks each tugboat driver participates in does not differ significantly from other tugboat drivers. Most literature considers the first two principles^6,7^, and this research will simultaneously consider the above three principles for the dispatch of tugboats.

After a vessel enters the port for operation, it is possible to estimate the arrival time and berthing time of the vessel, but it is difficult to predict the departure time from the berth because the loading and unloading time is difficult to determine, so the departure time may fluctuate. Hence, we can only know the notification time and determine the end time of this departure task. For the situation where this random task arrives, the strategy that will be explored in this research is to maximize the buffer time of this dynamic task and to facilitate the actual deployment of tugboats. Furthermore, the speed of vessels berthing, disembarking, and loading and unloading cargo mainly depends on the coordination of various buildings, facilities, machinery, tools, and personnel resources at the dock, and has great uncertainty. Moreover, the historical data makes it difficult to predict all operational situations. This article uses fuzzy numbers to describe the operation time of each task. In addition, busy ports need to serve dozens or even hundreds of vessels every day. When the problem scale increases, the model built in this study is difficult to solve and has certain limitations. Therefore, this paper proposes a metaheuristic algorithm to solve it.

In summary, it is necessary to weigh various factors to make scientific and reasonable tugboat scheduling decisions for busy ports. This paper investigates the optimization problem of tugboat scheduling to assist vessels in entering and exiting ports in uncertain environments and establishes a game-theoretic fuzzy model for tugboat scheduling in port operations to solve the trade-off problem between tugboat operators and port dispatchers in the tugboat scheduling process. In addition, this study incorporates three commonly used scheduling rules, task random arrival, and fuzziness into the modeling and develops a seagull optimization algorithm that sets priority encoding, fitness function, and genetic operators based on the characteristics of the tugboat scheduling problem addressed herein. The main purpose of this study is to assist tugboat operators and port dispatchers in making daily real-time decisions in vessel scheduling.

The innovation of this research lies in (1) considering the diversity of tugboat matching schemes to increase the optimality of tugboat scheduling; (2) taking into consideration of actual operational situations to make the model closer to reality; (3) using the principle of fair dispatching for tugboat scheduling; (4) exploring the situations where the task can arrive at any time; and (5) identifying the impact of game-theoretic decision-making on tugboat scheduling plans for the first time. The rest of this study is organized as follows. Section “Related work” reviews and summarizes the relevant work in the area of tugboat scheduling. Section “Problem description and modeling” provides the background of the problem and describes the composition of the model. Section Solution method outlines the main steps of the proposed solution algorithm. Section “Numerical experiments” formally presents the numerical experiments, and section “Conclusion” summarizes the results of this research.

## Related work

Some scholars have studied the optimization of tugboat resources from the perspective of classic allocation problems^8–12^. In the field of maritime transportation research, scholars have also conducted extensive research on the decision-making problems of berths or terminals, but there are few studies related to the optimization of tugboat resource allocation^12–14^. Early research often used heuristic algorithms to solve tugboat resource optimization problems. As an example, Wang and Meng^15^ developed a heuristic algorithm that combined a genetic algorithm and ant colony optimization for container terminal task resource allocation problems. In the first stage, the genetic algorithm used strings to represent the chromosomes of the allocation plan and found the optimal allocation through self-learning. In the second stage, an improved ant colony optimization algorithm was introduced to optimize scheduling tasks based on the genetic algorithm allocation plans. Finally, the performance of the algorithm was tested using a tugboat allocation problem, and satisfactory results were obtained.

In 2012, Wang et al.^16–18^ proposed a tugboat allocation optimization model from the perspective of matching the captain with the horsepower and quantity of tugboats and applied an improved trust-based ant colony optimization method to obtain the optimal scheduling plan. In the same year, they added time logic constraints to the model and established a mixed integer programming model that combined scheduling rules. They then provided a method to determine the optimal solution of the problem and analyzed the impact of the number of tugs and service capacity on ship turnover time through numerical experiments. However, the models in these three papers could not be directly solved until 2018, when Zhen et al.^19^ studied the tugboat allocation problem in seaports located at estuaries and proposed a mixed integer programming model to optimize the allocation scheme of barges to tugboats. They also proposed an accurate solution approach based on the branch-and-bound method to solve the proposed model. Wu et al.^20^ studied the evacuation planning problem of barges that may arise in reclamation projects. The authors made decisions from strategic, tactical, and operational perspectives on the allocation and scheduling of tugs for a team of heterogeneous barges working in offshore land reclamation areas that need to evacuate to coastal shelters before a storm arrives and proposed a customized heuristic method.

In terms of incorporating time slot resource allocation into the allocation of tugboats, Omar et al.^5^ considered the berth allocation problem with all required resources (tugs, pilots, pilot vessels, and mooring teams), safety considerations (within and between vessels), and berth location availability. They proposed a mixed integer programming model and precise solution method based on the constraint separation strategy for port entry. It was found that the decision on the start and end times of departure and relocation showed significant improvements in the management of port resources, especially during congestion periods. Jia et al.^21^ determined the schedule for tugs to serve vessels and the schedule for vessels to berth or depart during berthing and turning operations. A case study was considered based on the operational data from a container port in Shanghai to demonstrate that the proposed iterative algorithm combining Lagrange relaxation and Benders decomposition outperformed the benchmark algorithm in terms of solution quality. For scheduling operations in canals, Petris et al.^22^ took the medium-sized Port of Venice in Italy as an example and proposed four heuristic algorithms. The developed solution algorithms were found to be efficient and could provide high-quality solutions for most of the instances in a reasonable computational time.

Liu et al.^23^ proposed a two-stage stochastic mixed integer linear programming model for harbor berth and channel planning, considering the limited availability of berths and channels. The aim was to minimize the expected total weighted completion time of vessels in the presence of uncertainty in vessel arrival time and vessel processing duration. The first stage determined the allocation of berths for vessels under multi-scenario conditions. In the second stage, channel planning, including channel selection, tugboat allocation, and vessel sequencing, was determined in the presence of uncertainty. The authors proposed a stage decomposition method and decomposition-based heuristic algorithm to solve the problem. Kasm et al.^24^ established a mixed integer programming to simulate vessel scheduling under channel constraints and different tugboat allocation strategies and achieve better resource utilization. Jiang et al.^25^ established a mathematical optimization model from the perspective of time allocation, considering constraints, such as restricted channels and complex navigation rules for scheduling resources (including tugboats), and proposed an elite selection genetic algorithm encoded with vessel service sequences to solve the optimization model. It was pointed out that the modeling from the perspective of allocation issues required adding variables and increasing the complexity of the model.

Another group of scholars study tugboat scheduling optimization problems from the perspective of classic vehicle routing problems. For instance, Wei et al.^7^ formed a point line network of berths, tugboat bases, and artificial waypoints in defined port waters, establishing a mixed integer linear programming model for tugboat assisted container vessel berthing, moving, and unberthing processes, and developing six effective inequality groups that were incorporated into the standard branch-and-bound algorithm. However, this study simply considered that the tugboat base is a fixed starting and ending point, without considering that tugboats can dock at any tugboat base. In terms of rivers and seaports, Zhu et al.^26^ designed a variable neighborhood search algorithm to address the social responsibility of vesselping companies in reducing greenhouse gas emissions by arranging tugs and barges reasonably and determining the transportation route for barge transportation. The calculation results show that compared with the scheduling rules, the model reduced carbon emissions by approximately 46.93%. In addition, consideration of barge transportation in the model could reduce carbon emissions by approximately 10.46%. In addition, the variable neighborhood search algorithm demonstrated an optimality difference of approximately 0.29% in a short period. Hao et al.^27^ considered the joint scheduling of barges and tugs at river sea transfer ports with tidal influence channels, established an integer programming model based on a state spatiotemporal network to minimize the time required for bulk cargo transfer, and designed a customized variable neighborhood search algorithm. Wang et al.^28^ studied the tugboat scheduling problem considering multiple waypoints and multiple service modes, established a mixed integer linear programming model that can better utilize limited tugboats, and developed an efficient adaptive large neighborhood search algorithm. Li et al.^29^ proposed a new fuzzy programming optimization model for tugboat scheduling, considering multiple berthing bases, time windows, and operational uncertainties.

The remaining studies on tugboat scheduling optimization addressed the problem from the perspective of a flow shop. Kang et al.^30^ considered the uncertainty of container vessel arrival and tugboat process times in large container ports. The uncertain vessel arrival and tugboat operation process times were represented as a limited set of discrete scenarios, and uncertainty was addressed by integrating active and reactive scheduling strategies. The authors established a mixed integer linear programming model for the tugboat scheduling problem and designed a self-organizing algorithm to generate tugboat chains and solve large-scale problems. Although this study considered the uncertainty of vessel arrival and tugboat operation processes, the proposed approach would increase the difficulty of solving the model, and it is challenging to estimate the start time of an unpredictable operation for the vessel. Therefore, the present study adopted fuzzy numbers to replace the uncertainty of tugboat operation time and set a goal to reserve as much buffer time as possible for the operation to start. Taking into account natural factors, Zhong et al.^6^ studied the green tugboat scheduling problem for tidal ports with multiple port areas in long waterways. Considering the factor of the tidal time window, the schedule of tugboat service vessels was determined based on task sequence numbering and captured the trade-off between time and fuel consumption goals. The tidal time window was only a simple upper threshold value. However, modeling from the perspective of the flow workshop makes it difficult to address the issue of vessels not entering the port in the predetermined order.

Tug scheduling is a complex multi-party decision-making process, which has been studied by some scholars from the perspective of multi-stage decision-making. Musus et al.^31^ studied the strategic issue of locating and allocating tugs along the Norwegian coast to optimize sea readiness in emergency rescue and minimize the sum of the costs of public-operated tugs in emergency towing services and the expected penalty costs incurred due to inadequate preparation. A two-stage stochastic programming model was established, with the first stage positioning tugs to meet nominal coverage requirements, and the second stage deploying positioned tugs to assist vessels in distress. However, tugboats were allocated by port dispatchers and dispatched by barge operators, which can be described with a two-party game-theoretic process, and such a methodological approach is lacking in the existing literature.

In the process of tugboat dispatching, decision makers need to pay attention to fairness to balance the workload of tugboat drivers. Orgut et al.^32^ proposed by definition that each region within the service area should receive fairness constraints that are fully proportional to the needs of that region. The study also proposed mathematical models to promote fair and effective distribution of donated food by food banks among populations at risk of hunger. Se and Oyz^33^ studied the optimal distribution of influenza vaccines in heterogeneous populations composed of multiple subpopulations and proposed a mathematical model based on fairness constraints to help public health authorities incorporating fairness in vaccine allocation decisions. However, to the best of the authors’ knowledge, there are no significant research efforts on fair dispatch of tugboats.

In summary, the allocation and scheduling of tugboat resources is a relatively new topic. Although many researchers have devoted their studies to research certain aspects of the tugboat scheduling problem, there is little research on tugboat allocation and scheduling from the perspective of bipartite games, considering three commonly used scheduling rules, task random arrival, and fuzziness in relevant operational features. To this end, this study proposes a new mathematical model for optimizing the allocation and scheduling of tugboats from the perspective of a Stackelberg-based game-theoretic model, taking into account the well-known scheduling rules, task random arrival, allocation fairness, and fuzziness in operation times of tasks.

## Problem description and modeling

### Problem definition

Firstly, we clarify the main four definitions used in this study:

1. **Task**: Before the tugboat starts operations, the port scheduling party arranges the operation plan for the day through customer requests to receive the vessel’s operation requirements. As shown in Fig. 1, when a vessel enters the port, there are four processes on the port waters, including entering the channel, berthing, unberthing, and exiting the channel. Thus, vessels that require tugboat assistance in operation are generally divided into four tasks before entering the port. The service content, start time, and start and end locations of individual vessel operations can be known in advance, and the minimum number and minimum power of tugboats required can be determined based on the type of vessel. This study defines these four aspects of information as tasks. Specifically, when the port scheduling party makes berth plans, it is possible to predict the start time of vessel berthing, and the start time of entering the waterway can also be predicted through the length of the waterway. However, due to the difficulty in determining the loading and unloading time, the start time of departure is difficult to predict, and only the time and deadline for the task can be predicted. To this end, there are two types of tasks defined in this article: static tasks (i.e., tasks 1 and 2) and dynamic tasks (i.e., tasks 3 and 4). In addition, the task content also includes top flow, downstream turning anchoring, and anchor turning operations.
  **Fig. 1:**
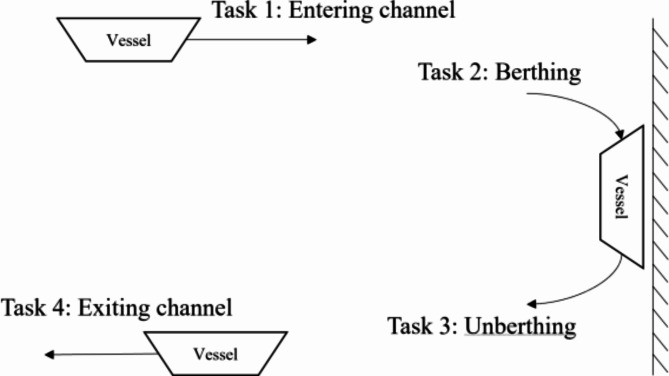
Entry and exit processes of a single vessel.
2. **Tugboat operation**: When working as a tugboat in a port and completing a given task, if there is no assignment for the next task available, the tugboat needs to proceed to a certain location within the port serving as a tugboat mooring base. If there is an assignment to handle the next task available, the tugboat needs to immediately sail to the location of the next task.
3. **Initial state**: It refers to the base where the tugboat docked after the previous day’s task was completed.
4. **Scheduling plan**: This refers to arranging tugboat services for each task based on the initial status of each tugboat and the set of tasks for a given day.

**Fig. 2:**
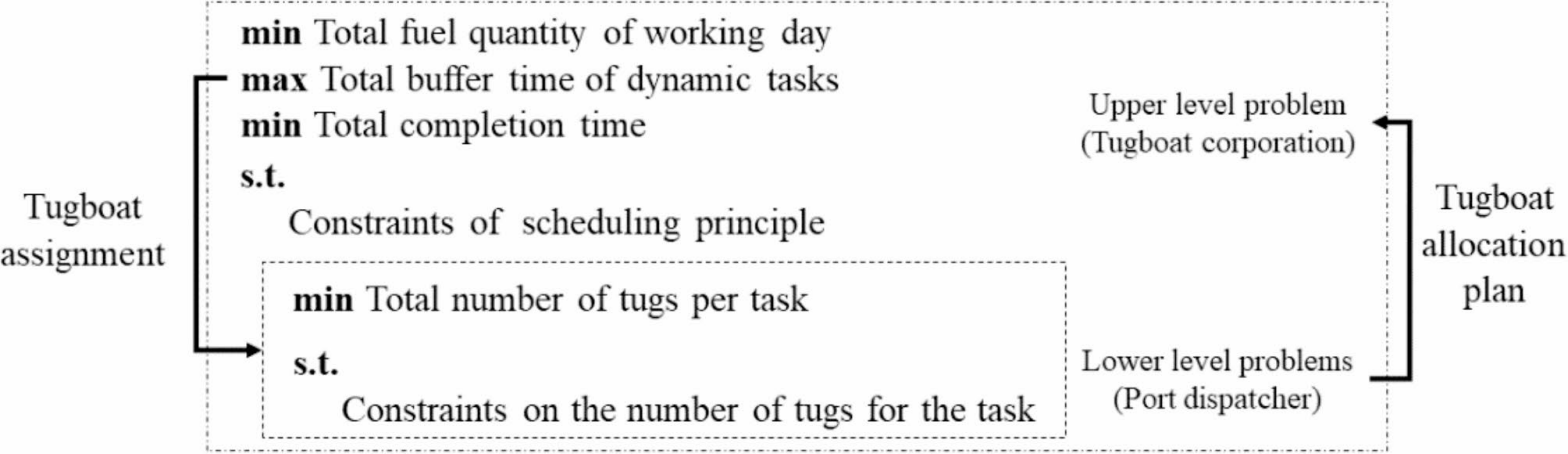
Schematic diagram of the Stackelberg game-theoretic model structure.

The tugboat scheduling problem studied in this paper is the allocation and scheduling problem between multiple berthing bases, multiple tugboats, and multiple tasks. The tugboat can dock at any berthing base but must start from a certain base (initial state), complete a task, return to a certain base, or continue to complete subsequent tasks. As shown in Fig. 2, considering the scarcity of tugboat resources in the port, the port scheduling party allocates a tugboat matching plan with the minimum number of tugboats for each task according to the “Standard for the Allocation of Tug Ships for Berthing, Leaving, Piloting, or Moving”, and then feeds back the plan to the tugboat operator’s scheduling plan. The tugboat operator can make decisions based on the status of the tugboat and the principle of shortest distance tugboat. The first available tugboat principle and the principle of fairness in the task volume of each tugboat are limited and implemented for dispatching from the location where the previous task was completed to the mooring base or dispatching a tugboat to the current task that needs to be executed. Hence, the decisions of both parties interact and form a game-theoretic relationship.

Tugboat services are charged based on the number of tugboat operations, and the total amount of fees is also determined after the task is determined. The quality of the scheduling plan depends on the level of operation costs, which are mainly based on fuel costs. Therefore, the goal of this study is to minimize the total fuel consumption, which consists of the driving fuel consumption (calculated by time) from the base to the start of the task, from the end of the task to the base, and from the end of the preceding task to the start of the succeeding task, as well as the operating fuel consumption (calculated by time) to complete the task. For dynamic tasks, the tugboat operator must also reserve enough time to dispatch suitable tugboats to serve them, while also taking into account the total tugboat operation time of the day, which could be affected by uncertainties. In the actual operational environment, due to the interference of uncertain factors, dispatchers cannot accurately grasp the port environment and operational changes. Therefore, the operation time of the task set in this study is uncertain, and the changes in parameters are represented by symmetric trapezoidal fuzzy numbers (as discussed more in detail in the following sections of the manuscript).

Based on the above description, the main decision problem studied in this research is the game-theoretic optimization process between the allocation of tugs by the port dispatcher and the dispatch of tugs by the tugboat operator. In the environment where dynamic tasks and fuzzy numbers coexist, the goal is to utilize the existing tug resources of tugboat operators to more economically and timely complete the operations involved in vessel entry and exit from the port.

### Model establishment

#### Assumptions

Based on the problem description, this study proposes the following assumptions: (1) the scheduling plan period is 1 day; (2) Each tugboat docks at the base before all tasks begin on that day; (3) The difference between external and internal berths of the base is not considered; (4) The standby time of the tugboat is short, and the time it takes to dock and leave the base berth is negligible; (5) The tugboat has a fixed speed (which is equal to the average speed); and (6) There is no limit to the number of tugboats that can be docked at each base.

#### Symbol description

For the convenience of establishing the model, this article defines the following symbols:

1. Set:
  *I* is the set of tasks,$i,j \in I=\{ 1,2, \ldots NI\}$,$NI$is total task amount;$I^{\prime}$is the set of dynamic tasks,$i,j \in I^{\prime},I^{\prime} \subseteq I$; *J* is the union set of 0 tasks and other tasks,$i,j \in J=\{ 0,1,2, \ldots ,NI\}$, 0 represents the task at 0 o’clock before the start of the day, which means that the task is not executed and a tugboat is only docked at the base; *K* is the set of tugboats,$k \in K=\{ 1,2, \ldots NK\}$,$NK$is the total number of tugboats; *L*is the set of bases,$l \in L=\{ 1,2, \ldots ,NL\}$,$NL$is the total amount of the bases; *R* is the union set of 0 base and other berthing bases,$l \in R=\{ 0,1,2, \ldots ,NL\}$, 0 means no docking at the base。
2. Parameters:
  ${\tilde {w}_i}$is the fuzzy service time for task *i*, $i \in J$, measured in min;$c_{{li}}^{1}$is the distance from base *l* to the starting point of task *i*,$l \in L,i \in J$, measured in km; $c_{{li}}^{2}$is the distance from base *l* to the end of task *i*,$l \in L,i \in J$, measured in km;${B_{kl}}$is the position of tugboat *k*at base *l*at the end of the previous day’s mission,$k \in K,l \in R$, which is a 0–1 matrix, if the tugboat stops at the base position, it is 1, otherwise it is 0; *M*is a sufficiently large value;${D_i}$is the number of tugboat requirement for task *i*,$i \in I$;$vc$is the average speed of the tugboat, measured in km/h;${P_k}$is the power of tugboat *k*, $k \in K$, measured in HP;${m_i}$is the required tugboat horsepower for task *i*,$i \in I$, measured in HP;${\gamma _k}$is the fuel consumption per unit time of operation,$k \in K$, measured in kg/min;$C{o_k}$is the unit fuel consumption during navigation,$k \in K$, measured in kg/min;${Q_i}$is the number of tugboats with specified power requirements for task *i*,$i \in I$;$c_{{ij}}^{3}$is the distance from the end point of task *i*to the starting point of task *j*,$i,j \in I$, measured in km;${A_i}$is the estimated start time of task *i*,$i \in I$, specifically, when$i \in I^{\prime}$, this symbol represents the time when the notification for task *i*exists, measured in min;${\tau _i}$is the maximum waiting time for task *i*,$i \in I$, specifically, when$i \in I^{\prime}$, this symbol represents the deadline period for task *i*, measured in min;${\tilde {\pi }_i}$is the fuzzy maximum delay time of tugboat service for task *i*,$i \in I$, measured in min; *N* is the fairness coefficient, with the value of$\left[ {0,1} \right]$.
3. Upper level decision variables:
  ${x_{klij}}$ is a 0–1 variable, and if the tugboat *k*completes task *i*and returns to base *l*to complete task *j*, it is 1; otherwise, it is 0, $k \in K,l \in R,i,j \in J$;${s_{ki}}$is the start time of task *i*served by tugboat *k*,$k \in K,i \in J$;${y_{ki}}$is the service time of tugboat *k*for task *i*,$k \in K,i \in J$.
4. Lower level decision variables:
  ${x^{\prime}_{ki}}$is a 0–1 variable, if tugboat *k*is assigned to task *i*, it is 1; otherwise, it is 0,$k \in K,i \in I$.

#### Upper level programming model

The upper-level decision maker is the tugboat operator, whose goal is to reasonably dispatch tugboats to carry out the tasks, to minimize the daily total fuel consumption of their company, and to refer to the requirements of the port’s tugboat matching plan. The upper-level planning model M1 has three objective functions, namely minimizing the total daily fuel consumption of tugboat operations, maximizing the total buffer time of dynamic tasks, and minimizing the total completion time:

1. Objective function 1 (total fuel consumption for tugboat operations) includes minimizing the sum of fuel consumption when sailing from the base to the start point of the task, from the end point of the task to the base, from the end point of the previous task to the start point of the next task, and the waiting time from the start time of the task to the end time of the task:
  1 $$\begin{gathered} \hbox{min} \;{f_1}=60\sum\limits_{{k \in K}} {\sum\limits_{{l \in L}} {\sum\limits_{{i \in J}} {\sum\limits_{{j \in I}} {{{C{o_k}c_{{lj}}^{1}{x_{klij}}} \mathord{\left/ {\vphantom {{C{o_k}c_{{lj}}^{1}{x_{klij}}} {vc}}} \right. \kern-0pt} {vc}}} } } } +60\sum\limits_{{k \in K}} {\sum\limits_{{l \in L}} {\sum\limits_{{i \in I}} {\sum\limits_{{j \in J}} {{{C{o_k}c_{{li}}^{2}{x_{klij}}} \mathord{\left/ {\vphantom {{C{o_k}c_{{li}}^{2}{x_{klij}}} {vc}}} \right. \kern-0pt} {vc}}} } } } \hfill \\ \quad \quad \quad \quad +60\sum\limits_{{k \in K}} {\sum\limits_{{l \in \left\{ 0 \right\}}} {\sum\limits_{{i \in I}} {\sum\limits_{{j \in I}} {{{C{o_k}c_{{ij}}^{3}{x_{klij}}} \mathord{\left/ {\vphantom {{C{o_k}c_{{ij}}^{3}{x_{klij}}} {vc}}} \right. \kern-0pt} {vc}}} } } } +\sum\limits_{{k \in K}} {\sum\limits_{{i \in I}} {{\gamma _k}{y_{ki}}} } \hfill \\ \end{gathered}$$
2. Objective function 2 (total buffer time of dynamic tasks) is expressed as maximizing the sum of the difference between the start time of the dynamic task and the end time of the immediately preceding task:
  2 $$\hbox{max} \;{f_2}=\sum\limits_{{k \in K}} {\sum\limits_{{l \in L}} {\sum\limits_{{i \in I}} {\sum\limits_{{j \in I^{\prime}}} {\left[ {\left( {{A_j}+{\tau _j} - {y_{kj}}} \right){x_{klij}} - \left( {{s_{ki}}+{y_{ki}}} \right){x_{klij}}} \right]} } } }$$
3. Objective function 3 (total completion time) represents minimizing the maximum task end time:
  3 $$\hbox{min} \;{f_3}=\mathop {\hbox{max} }\limits_{{k \in K,i \in I}} \left\{ {{s_{ki}}+{y_{ki}}} \right\}$$

According to the model assumptions, the decision variables need to meet the following constraints:

4 $$\sum\limits_{{j \in J}} {{x_{kl0j}}} \leqslant {B_{kl}},\quad \forall k \in K,l \in R$$

5 $$\sum\limits_{{l \in R}} {\sum\limits_{{i \in J}} {{x_{klih}}} } - \sum\limits_{{l \in R}} {\sum\limits_{{j \in J}} {{x_{klhj}}} } =0,\quad \forall h \in I,k \in K$$

6 $${s_{ki}}+{y_{ki}}+{{60 \times {x_{klij}}c_{{li}}^{2}} \mathord{\left/ {\vphantom {{60 \times {x_{klij}}c_{{li}}^{2}} {vc}}} \right. \kern-0pt} {vc}}+60 \times {{{x_{klij}}c_{{lj}}^{1}} \mathord{\left/ {\vphantom {{{x_{klij}}c_{{lj}}^{1}} {vc}}} \right. \kern-0pt} {vc}} - M\left( {1 - {x_{klij}}} \right) \leqslant {s_{kj}},\quad \forall i,j \in I,k \in K,l \in L$$

7 $${s_{ki}}+{y_{ki}}+{{60 \times c_{{ij}}^{3}{x_{klij}}} \mathord{\left/ {\vphantom {{60 \times c_{{ij}}^{3}{x_{klij}}} {vc}}} \right. \kern-0pt} {vc}} - M\left( {1 - {x_{klij}}} \right) \leqslant {s_{kj}},\quad \forall i,j \in I,k \in K,l \in \left\{ 0 \right\}$$

8 $${A_i}{x^{\prime}_{ki}} \leqslant {s_{ki}} \leqslant \left( {{A_i}+{\tau _i}} \right){x^{\prime}_{ki}},\quad \forall k \in K,i \in I$$

9 $${\tilde {w}_i}{x^{\prime}_{ki}} \leqslant {y_{ki}} \leqslant \left( {{{\tilde {w}}_i}+{{\tilde {\pi }}_i}} \right){x^{\prime}_{ki}},\quad \forall k \in K,i \in I$$

10 $${s_{ki}}+{y_{ki}} \leqslant \left( {{A_i}+{\tau _i}} \right){x^{\prime}_{ki}},\quad \forall k \in K,i \in I^{\prime}$$

11 $${x_{klij}}=0,\quad \forall k \in K,l \in \left\{ 0 \right\},i \in J,j \in \left\{ 0 \right\}$$

12 $$\left| {\frac{{\sum\limits_{{i \in I}} {{{x^{\prime}}_{ki}}} }}{{\sum\limits_{{k^{\prime} \in K}} {\sum\limits_{{i \in I}} {{{x^{\prime}}_{k^{\prime}i}}} } }} - \frac{{\left\lfloor {{{\sum\limits_{{i \in I}} {{D_i}} } \mathord{\left/ {\vphantom {{\sum\limits_{{i \in I}} {{D_i}} } {NK}}} \right. \kern-0pt} {NK}}} \right\rfloor }}{{\sum\limits_{{i \in I}} {{D_i}} }}} \right| \leqslant N,\quad \forall k \in K$$

13 $${x_{klij}},{x^{\prime}_{ki^{\prime}}} \in \left\{ {0,1} \right\},\quad \forall k \in K,l \in L,i^{\prime} \in I,i,j \in J$$

14 $${s_{ki}},{y_{ki}} \geqslant 0,\quad \forall k \in K,i \in J$$

Among them, Eq. (4) ensures that each tugboat is positioned at the initial mooring base after completing the tasks from the previous day. Equation (5) assures that a given tugboat is dispatched to the next task after completing the previous task. Equation (6) indicates that the start and execution times of the previous task, the total time to return to a certain base after completing this task, and then depart from this base to reach the starting position of the next task should be earlier than the start operation time of the next task. Equation (7) ensures that the start and execution times of the previous task, and the total time required to directly reach the starting position of the next task should be earlier than the start operation time of the next task. Equation (8) limits the task start time to a certain range. Equation (9) limits the service time to a certain allowable range. Equation (10) imposes constraints on the actual dynamic task end time based on the maximum task waiting time. Equation (11) restricts a given tugboat from stopping at non-zero base at the end of the day. Equation (12) represents a fair constraint on the task quantity for each tugboat, which adjusts the fairness of the task quantity assigned to the tugboat by changing the value of N. Equation (13) represents the integrality constraints for the 0–1 variables; Eq. (14) represents the integrality constraints for the variables that are not less than zero.

Moreover, when *N* is 0, to maintain the feasibility of Eq. (12), Eq. (12) can be replaced with Eq. (15) that can be formulated as follows:

15 $$\sum\limits_{{i \in I}} {{{x^{\prime}}_{ki}}} \geqslant \left\lfloor {{{\sum\limits_{{i \in I}} {{D_i}} } \mathord{\left/ {\vphantom {{\sum\limits_{{i \in I}} {{D_i}} } {NK}}} \right. \kern-0pt} {NK}}} \right\rfloor ,\quad \forall k \in K$$

#### Lower level programming model

As a scarce resource, tugboats need to be matched according to the tugboat matching principle. The port side of the lower-level decision maker aims to minimize the total number of different types of tugboats matched for each task and obtain the optimal tugboat matching scheme for each task, which will be fed back to the tugboat scheduling plan of the upper-level decision maker. The planning model M2 can be formulated as follows:

16 $${x^{\prime}_{ki}} \in \arg \;\mathop {\hbox{min} }\limits_{{i \in I}} \;\sum\limits_{{k \in K}} {{{x^{\prime}}_{ki}}}$$

17 $$\sum\limits_{{k \in K}} {{{x^{\prime}}_{ki}}} \geqslant {D_i}:{\lambda _i},\quad \forall i \in I$$

18 $$\left\{ \begin{gathered} \sum\limits_{{k \in \left\{ {o|{P_o} \geqslant {m_i},o \in K} \right\}}} {{{x^{\prime}}_{ki}}} \geqslant {Q_i}:\mu _{i}^{1},\quad \forall i \in I \hfill \\ or \hfill \\ \sum\limits_{{k \in K}} {{P_k}{{x^{\prime}}_{ki}}} >{m_i}{Q_i}:\mu _{i}^{2},\quad \forall i \in I \hfill \\ \end{gathered} \right.$$

19 $${x^{\prime}_{ki}}=\sum\limits_{{l \in R}} {\sum\limits_{{j \in J}} {{x_{klij}}} } :{\eta _{ki}},\quad \forall k \in K,i \in I$$

20 $${x_{klij}},{x^{\prime}_{ki}} \in \left\{ {0,1} \right\},\quad \forall k \in K,l \in L,i,j \in J$$

Wherein, Eq. (16) aims to minimize the total number of tugboats for the available tasks. Equation (17) ensures the number of tugboats required is allocated to serve a given task, and ${\lambda _i}$ is the dual variable of this constraint condition. For a task, according to the requirements of the port, a total of *D* tugboats may be necessary, among which *Q*tugboats with *P*horsepower can work together to complete the tugboat auxiliary phase. However, sometimes, as long as the total power of the tugboat serving the task is greater than the$Q \times P$horsepower, the power supply demand can also be met. Therefore, Eq. (18) restricts the number of tugboats with rated horsepower required for a given task to not be lower than the minimum required for the task or to require horsepower greater than the total rated horsepower.$\mu _{i}^{1}$and$\mu _{i}^{2}$represent the dual variables of each constraint condition. Equation (19) represents the relationship between upper and lower decision variables, with${\eta _{ki}}$being the dual variable of the constraint condition. Equation (20) indicates that the main variables are 0–1 variables.

### Bi-level model framework

#### Stackelberg game

Generally speaking, the Stackelberg game planning problem involving two players can be characterized using a bilevel programming model. Because in the game-theoretic process, the dominant player makes decisions first, and the upper level of the model takes the dominant player as the decision-making subject along with its decision variables. The follower makes decisions after the leader, so it corresponds to the lower-level optimization problem and its decision variable. Due to the sequential nature of decision-making, for the lower-level model, the dominant decision variable is considered as the known parameter that is passed from the upper level. In addition, in the proposed optimization framework, the upper-level model imposes constraints, reflecting the impact of follower decision-making on the leader. Based on this, it can be seen that the bilevel programming model can well reflect the mutual constraint relationship between the leader and follower in the Stackelberg game planning process. The general expression for the above Stackelberg game-theoretic problem can be described using the bilevel programming model (21).

21 $$\begin{gathered} \mathop {\hbox{min} }\limits_{{\left\{ x \right\} \cup \left\{ {y,\lambda ,\mu } \right\}}} F\left( {x,y,\lambda ,\mu } \right) \hfill \\ s.t.\quad H\left( {x,y,\lambda ,\mu } \right)=0, \hfill \\ \quad \quad G\left( {x,y,\lambda ,\mu } \right) \geqslant 0, \hfill \\ \quad \quad \left\{ \begin{gathered} \mathop {\hbox{min} }\limits_{y} \;f\left( {x,y} \right) \hfill \\ s.t.\;\;h\left( {x,y} \right)=0:\lambda , \hfill \\ \quad \;\;g\left( {x,y} \right) \geqslant 0:\mu , \hfill \\ \end{gathered} \right. \hfill \\ \end{gathered}$$

Where$F\left( {x,y,\lambda ,\mu } \right)$and$f\left( {x,y} \right)$are the objective functions for the upper- and lower-level optimization problems, respectively.$h\left( {x,y} \right)=0$is the equality constraint of the lower-level optimization problem, and its corresponding dual variable is$\lambda$.$g\left( {x,y} \right) \geqslant 0$is the inequality constraint of the lower-level optimization problem, whose corresponding dual variable is$\mu$. $H\left( {x,y,\lambda ,\mu } \right)=0$and$G\left( {x,y,\lambda ,\mu } \right) \geqslant 0$ are the equality and inequality constraints for the upper-level optimization problem, respectively. The optimization variable of the lower level model is the decision variable of the follower. The optimization variables of the upper model include not only the decision variable *y* of the leader, but also the optimization variable *x* of the lower model, as well as the dual variables of the lower model.

Stackelberg game-theoretic models are very different from traditional optimization models and require specific solution approaches. At present, the commonly used algorithms for solving Stackelberg game-theoretic models include the following^34^: (1) the Karush–Kuhn–Tucker (KKT) optimality condition method, which uses the KKT optimality conditions to replace the lower-level decision problem, thereby transforming the hierarchical game-theoretic model into traditional linear programming; (2) the Penalty function method, which converts the original problem into a single-layer optimization problem through the principle of penalty function; (3) Descending direction method, which utilizes the commonly used steepest descent method and rotation scale-based methods in nonlinear programming to find local or global optimal solutions; (4) Global optimization method, which converts the original problem into a concave minimization problem and uses global optimization methods to solve it. In addition, some intelligent optimization algorithms and metaheuristics, such as particle swarm optimization, are also used to solve Stackelberg game-theoretic models. This article uses the KKT optimality condition method to solve the Stackelberg game-theoretic model (21).

If the lower level model is a linear programming problem, it can be equivalently replaced with the corresponding KKT condition, and then the original bilevel model can be transformed into a single-layer optimization problem, such as model (22).

22 $$\begin{gathered} \mathop {\hbox{min} }\limits_{{x,y,\lambda ,\mu }} F\left( {x,y,\lambda ,\mu } \right) \hfill \\ s.t.\quad \quad \left\{ \begin{gathered} H\left( {x,y,\lambda ,\mu } \right)=0, \hfill \\ G\left( {x,y,\lambda ,\mu } \right) \geqslant 0, \hfill \\ {\nabla _y}f\left( {x,y} \right) - {\lambda ^T}{\nabla _y}h\left( {x,y} \right) - {\mu ^T}{\nabla _y}g\left( {x,y} \right)=0, \hfill \\ h\left( {x,y} \right)=0, \hfill \\ 0 \leqslant \mu \bot g\left( {x,y} \right) \geqslant 0 \hfill \\ \end{gathered} \right. \hfill \\ \end{gathered}$$

Wherein,$$0 \leqslant \mu \bot g\left( {x,y} \right) \geqslant 0\; \Leftrightarrow \;\mu \geqslant 0,g\left( {x,y} \right) \geqslant 0,\mu \leqslant Mz,g\left( {x,y} \right) \leqslant M\left( {1 - z} \right)$$,$$z \in \left\{ {0,1} \right\}$$.

At this point, the original bilevel programming model can be transformed into a mixed integer linear programming problem as follows:

23 $$\begin{gathered} \mathop {\hbox{min} }\limits_{{x,y,\lambda ,\mu }} F\left( {x,y,\lambda ,\mu } \right) \hfill \\ s.t.\quad \quad \left\{ \begin{gathered} H\left( {x,y,\lambda ,\mu } \right)=0, \hfill \\ G\left( {x,y,\lambda ,\mu } \right) \geqslant 0, \hfill \\ {\nabla _y}f\left( {x,y} \right) - {\lambda ^T}{\nabla _y}h\left( {x,y} \right) - {\mu ^T}{\nabla _y}g\left( {x,y} \right)=0, \hfill \\ h\left( {x,y} \right)=0, \hfill \\ 0 \leqslant \mu \leqslant M\left( {1 - z} \right), \hfill \\ 0 \leqslant g\left( {x,y} \right) \leqslant Mz, \hfill \\ z \in \left\{ {0,1} \right\} \hfill \\ \end{gathered} \right. \hfill \\ \end{gathered}$$

The optimization model proposed in this study can be solved using the aforementioned method, that is, the lower-level linear programming with the KKT optimality conditions (reserved Eq. (19)):

24 $$0 \leqslant {\lambda _i} \leqslant M\left( {1 - z_{i}^{1}} \right),\quad \forall i \in I$$

25 $$0 \leqslant \sum\limits_{{k \in K}} {{{x^{\prime}}_{ki}}} - {D_i} \leqslant Mz_{i}^{1},\quad \forall i \in I$$

26 $$\begin{gathered} \left\{ \begin{gathered} 1 - {\eta _{ki}} - {\lambda _i} - \mu _{i}^{1}=0,\quad \forall k \in K,i \in I \hfill \\ 0 \leqslant \mu _{i}^{1} \leqslant M\left( {1 - z_{i}^{2}} \right),\quad \forall i \in I \hfill \\ 0 \leqslant \sum\limits_{{k \in \left\{ {o|{P_o} \geqslant {m_i},o \in K} \right\}}} {{{x^{\prime}}_{ki}}} - {Q_i} \leqslant Mz_{i}^{2},\quad \forall i \in I \hfill \\ \end{gathered} \right. \hfill \\ or \hfill \\ \left\{ \begin{gathered} 1 - {\eta _{ki}} - {\lambda _i} - \mu _{i}^{2}=0,\quad \forall k \in K,i \in I \hfill \\ 0 \leqslant \mu _{i}^{2} \leqslant M\left( {1 - z_{i}^{3}} \right),\quad \forall i \in I \hfill \\ 0<\sum\limits_{{k \in K}} {{P_k}{{x^{\prime}}_{ki}}} - {m_i}{Q_i} \leqslant Mz_{i}^{3},\quad \forall i \in I \hfill \\ \end{gathered} \right. \hfill \\ \end{gathered}$$

27 $$z_{i}^{1},z_{i}^{2},z_{i}^{3} \in \left\{ {0,1} \right\},\quad \forall i \in I$$

#### Fuzzy parameters

Many scholars use fuzzy theory to deal with uncertainty problems, such as Huang et al.^35^ using a compromise fuzzy programming method to construct regional sewage system planning. However, according to the study conducted by Dehshiri et al.^36^ and their proposed definition of the *Me* measure, the following equivalent formula can be derived:

28 $$Me\left\{ {\tilde {\xi } \leqslant x} \right\} \geqslant \alpha \Leftrightarrow \lambda ^{\prime}+(1 - \lambda ^{\prime})\frac{{x - {\xi ^c}}}{{{\xi ^d} - {\xi ^c}}} \geqslant \alpha \Leftrightarrow x \geqslant \frac{{(\alpha - \lambda ^{\prime}){\xi ^d}+(1 - \alpha ){\xi ^c}}}{{1 - \lambda ^{\prime}}}$$

29 $$Me\left\{ {\tilde {\xi } \geqslant x} \right\} \geqslant \alpha \Leftrightarrow \lambda ^{\prime}+(1 - \lambda ^{\prime})\frac{{{\xi ^b} - x}}{{{\xi ^b} - {\xi ^a}}} \geqslant \alpha \Leftrightarrow x \leqslant \frac{{(\alpha - \lambda ^{\prime}){\xi ^a}+(1 - \alpha ){\xi ^b}}}{{1 - \lambda ^{\prime}}}$$

If$\lambda ^{\prime}$ = 1, then Me = Pos (where Pos is the possibility measure), indicating that the decision maker considers the possibility measure and uses the maximum probability. If$\lambda ^{\prime}$ = 0, then Me = Nec (where Pos is the necessity measure), indicating that the decision maker considers the necessity measure and uses the minimum probability; If$\lambda ^{\prime}$ = 0.5, then Me = Cr, the decision maker considers the feasibility measure and uses the average probability.

Based on the above analysis, Eq. (9) can be replaced by Eqs. (30) and (31) as follows:

30 $$\frac{{(\alpha - \lambda ^{\prime})w_{i}^{d}+(1 - \alpha )w_{i}^{c}}}{{1 - \lambda ^{\prime}}}{x^{\prime}_{ki}} \leqslant {y_{ki}},\quad \forall k \in K,i \in I$$

31 $${y_{ki}} \leqslant \frac{{(\beta - \lambda ^{\prime})(w_{i}^{a}+\pi _{i}^{a})+(1 - \beta )(w_{i}^{b}+\pi _{i}^{b})}}{{1 - \lambda ^{\prime}}}{x^{\prime}_{ki}},\quad \forall k \in K,i \in I$$

Wherein, $\alpha$ and $\beta$ are the confidence levels.

#### Transformation of the objective functions

The upper-level programming model is a multi-objective programming model that could be challenging to solve directly. Therefore, the appropriate steps need to be taken to convert the multi-objective model into a single-objective model, which include the following.

**Step 1**: Linearize objective function 3.

Objective function 3 can be updated as follows:

32 $$\hbox{min} \;{f_3}={C_{\hbox{max} }}$$

And a new inequality should be added in the optimization model:

33 $${s_{ki}}+{y_{ki}} \leqslant {C_{\hbox{max} }},\quad \forall k \in K,i \in I$$

**Step 2**: Linearize objective function 2.

Objective function 2 contains nonlinear components (multiplication of two variables). Let $Y_{{klij}}^{1}={y_{kj}}{x_{klij}}$,$Y_{{klij}}^{2}={s_{ki}}{x_{klij}}$and$Y_{{klij}}^{3}={y_{ki}}{x_{klij}}$, then objective function 2 can be repressed as follows:

34 $$\hbox{max} \;{f_2}=\sum\limits_{{k \in K}} {\sum\limits_{{l \in L}} {\sum\limits_{{i \in I}} {\sum\limits_{{j \in I^{\prime}}} {\left[ {\left( {{A_j}+{\tau _j}} \right){x_{klij}} - Y_{{klij}}^{1} - Y_{{klij}}^{2} - Y_{{klij}}^{3}} \right]} } } }$$

New constraints should be added in the optimization model:

35 $$Y_{{klij}}^{1} \leqslant {y_{kj}},\quad \forall k \in K,l \in L,i \in I,j \in I^{\prime}$$

36 $$Y_{{klij}}^{1} \leqslant M \cdot {x_{klij}},\quad \forall k \in K,l \in L,i \in I,j \in I^{\prime}$$

37 $$Y_{{klij}}^{1} \geqslant M \cdot ({x_{klij}} - 1)+{y_{kj}},\quad \forall k \in K,l \in L,i \in I,j \in I^{\prime}$$

38 $$Y_{{klij}}^{2} \leqslant {s_{ki}},\quad \forall k \in K,l \in L,i \in I,j \in I^{\prime}$$

39 $$Y_{{klij}}^{2} \leqslant M \cdot {x_{klij}},\quad \forall k \in K,l \in L,i \in I,j \in I^{\prime}$$

40 $$Y_{{klij}}^{2} \geqslant M \cdot ({x_{klij}} - 1)+{s_{ki}},\quad \forall k \in K,l \in L,i \in I,j \in I^{\prime}$$

41 $$Y_{{klij}}^{3} \leqslant {y_{ki}},\quad \forall k \in K,l \in L,i \in I,j \in I^{\prime}$$

42 $$Y_{{klij}}^{3} \leqslant M \cdot {x_{klij}},\quad \forall k \in K,l \in L,i \in I,j \in I^{\prime}$$

43 $$Y_{{klij}}^{3} \geqslant M \cdot ({x_{klij}} - 1)+{y_{ki}},\quad \forall k \in K,l \in L,i \in I,j \in I^{\prime}$$

$$Y_{{klij}}^{1},Y_{{klij}}^{2},Y_{{klij}}^{3} \geqslant 0,\quad \forall k \in K,l \in L,i \in I,j \in I^{\prime}$$

**Step 3**: Convert the three objectives into a single objective function.

Firstly, define each objective function as a linear membership function:

44 $${\mu _1}\left( v \right)=\left\{ \begin{gathered} 1,\quad \quad \quad \quad \quad {f_1}<f_{1}^{{PIS}} \hfill \\ \frac{{f_{1}^{{NIS}} - {f_1}}}{{f_{1}^{{NIS}} - f_{1}^{{PIS}}}},\quad f_{1}^{{PIS}} \leqslant {f_1} \leqslant f_{1}^{{NIS}} \hfill \\ 0\quad \quad \quad \quad \quad {f_1}>f_{1}^{{NIS}} \hfill \\ \end{gathered} \right.$$

45 $${\mu _2}\left( v \right)=\left\{ \begin{gathered} 1,\quad \quad \quad \quad \quad {f_2}>f_{2}^{{PIS}} \hfill \\ \frac{{{f_2} - f_{2}^{{NIS}}}}{{f_{2}^{{PIS}} - f_{2}^{{NIS}}}},\quad f_{2}^{{NIS}} \leqslant {f_2} \leqslant f_{2}^{{PIS}} \hfill \\ 0\quad \quad \quad \quad \quad {f_2}<f_{2}^{{NIS}} \hfill \\ \end{gathered} \right.$$

46 $${\mu _3}\left( v \right)=\left\{ \begin{gathered} 1,\quad \quad \quad \quad \quad {f_3}<f_{3}^{{PIS}} \hfill \\ \frac{{f_{3}^{{NIS}} - {f_3}}}{{f_{3}^{{NIS}} - f_{3}^{{PIS}}}},\quad f_{3}^{{PIS}} \leqslant {f_3} \leqslant f_{3}^{{NIS}} \hfill \\ 0\quad \quad \quad \quad \quad {f_3}>f_{3}^{{NIS}} \hfill \\ \end{gathered} \right.$$

Where ${\mu _j}(v)$ represents the satisfaction of the *j*-th objective function.

In addition, according to the method proposed by Bilgen^37^, a payoff table can be used (see Table 1), where${f_t}(X)$is the *t*-th objective function and${X^{(t)}}$is its optimal solution, to ensure the feasibility of each objective range.

**Table 1:** Payoff table.

	f_1_(X)	f_2_(X)	…	f_t_(X)
*X* ^(1)^	*f*_11_	*f*_12_	…	*f*_1*t*_
*X* ^(2)^	*f*_21_	*f*_22_	…	*f*_2*t*_
…	…	…	…	…
*X* ^(*t*)^	*f*_*t*1_	*f*_*t*1_	…	*f*_*tt*_

The lower and upper bounds of the minimization and maximization problems can be determined from the matrix in Table 1 using Eqs. (47) and (48), respectively:

47 $$f_{t}^{{\hbox{min} }}=\hbox{min} \left\{ {{f_{1t}},{f_{2t}}, \ldots ,{f_{tt}}} \right\}$$

48 $$f_{t}^{{\hbox{max} }}=\hbox{max} \left\{ {{f_{1t}},{f_{2t}}, \ldots ,{f_{tt}}} \right\}$$

Then there are $f_{1}^{{PIS}}=\hbox{min} \left\{ {{f_{11}},{f_{21}},{f_{31}}} \right\}$,$f_{1}^{{NIS}}=\hbox{max} \left\{ {{f_{11}},{f_{21}},{f_{31}}} \right\}$,$f_{2}^{{PIS}}={\hbox{max} _2}\left\{ {{f_{12}},{f_{22}},{f_{32}}} \right\}$,$f_{2}^{{NIS}}=\hbox{min} \left\{ {{f_{12}},{f_{22}},{f_{32}}} \right\}$,$f_{3}^{{PIS}}=\hbox{min} \left\{ {{f_{13}},{f_{23}},{f_{33}}} \right\}$ and $f_{3}^{{NIS}}=\hbox{max} \left\{ {{f_{13}},{f_{23}},{f_{33}}} \right\}$.

Then, using the aggregation function of Torabi and Hassini^38^, the three objective functions can be transformed into the following formula:

49 $$\hbox{max} \;\psi {\lambda _0}+(1 - \psi )\left[ {{\theta _1}{\mu _1}(v)+{\theta _2}{\mu _2}(v)+{\theta _3}{\mu _3}(v)} \right]$$

The following constraints should be added in the optimization model:

50 $${\lambda _0} \leqslant {\mu _j}(v),\quad \forall j=\left\{ {1,2,3} \right\}$$

51 $${\lambda _0} \geqslant 0$$

Where${\lambda _0}=\mathop {\hbox{min} }\limits_{j} \left\{ {{\mu _j}(v)} \right\}$,$\psi$is the minimum satisfaction level for the objectives, $\psi \in \left[ {0,1} \right]$, ${\theta _j}$are the coefficients determined based on the decision maker’s preference, and$\sum\nolimits_{{j=1,2,3}} {{\theta _j}} =1,{\theta _j} \geqslant 0$.

In summary, the Stackelberg game-theoretic models M1 and M2 can be transformed into a single-objective mixed integer linear programming model M3 with Eq. (49) as the objective, equations from (4) to (8), equations from (10) to (14), Eq. (19), equations from (24) to (27), equations from (30) to (31), Eq. (33), equations from (35) to (46), and equations from (50) to (51) as constraints.

## Solution method

The model M3 constructed in the previous section is a mixed integer linear programming model, which can be directly solved using popular commercial solvers, such as CPLEX, LINGO, and GAMS. However, as the scale of the problem increases, the efficiency of existing exact optimization solvers becomes quite low. In this case, heuristic and metaheuristic algorithms, such as genetic algorithms, nested segmentation algorithm, and simulated annealing algorithm, can be used to improve the solving efficiency and obtain good-quality solutions in a short period of time.

The seagull optimization algorithm (SOA) is a new swarm intelligence optimization algorithm proposed by Dhiman and Kumar^39^, which mainly simulates the migration of seagulls in nature and their attack behavior (foraging behavior) during migration. This algorithm simulates the behavior of seagulls during migration and predation, constructs a mathematical model, and achieves the goal of finding good-quality solutions to the combinatorial optimization problem. After the model construction is completed, the stability and superiority of SOA are verified through a large number of test functions and comparative experiments. Finally, SOA has been introduced to solve large-scale industrial engineering problems, and its strong practicality has been verified using different industrial optimization problems. In addition, SOA has also been applied to a variety of decision problems, such as multi-reservoir systems^40^, cogeneration systems^41^, predictive models^42^, overcurrent relays^2^, and shield tunneling prediction^43^.

However, like other swarm intelligence optimization algorithms, SOA also has certain drawbacks, such as premature convergence, relatively weak global optimization ability, poor population diversity, and susceptibility to local optima. The main reason for this is the lack of communication between individuals in the seagull population in the SOA algorithm. Therefore, to overcome the existing limitations of the seagull optimization algorithm, this study utilizes genetic operators, such as crossover and mutation operations widely deployed in traditional genetic algorithms^44,45^, to increase the exchange of information between individuals, thus expanding the search scope of seagull groups and improving the search capability of SOA.

**Fig. 3:**
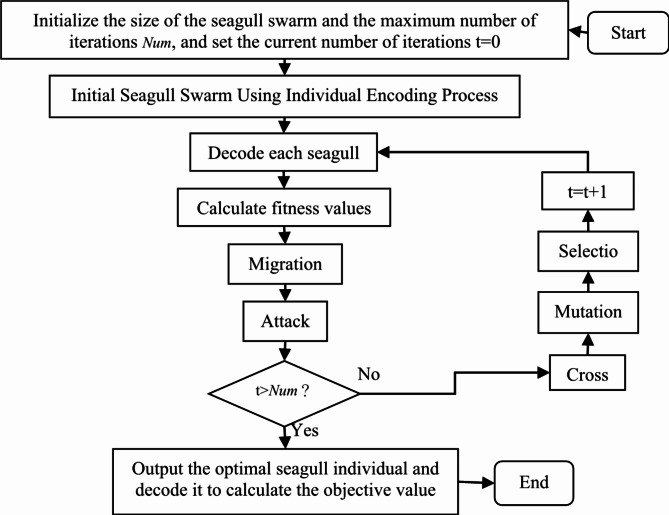
The main steps of the SOAPG algorithm.

The priority encoding method has been successfully applied in different optimization algorithms for solving large-scale strategic logistics network design problems^46–49^. This encoding method has the characteristics of simple operation and easy generation. Based on the characteristics of tugboat scheduling problems and the structure of priority encoding methods, this paper proposes a priority encoding scheme with mooring bases. In summary, this study presents a Seagull Optimization Algorithm based on Priority Encoding and Genetic Operators (SOAPG) to solve model M3. The algorithm flow is shown in Fig. 3.

### Individual encoding and decoding operations

#### Individual encoding operation

Considering that this study focuses on the allocation problem between tugboats, tasks, and docking bases, after studying the solution structure, it is necessary to determine which tugboats are serving each task, and whether a given tugboat will directly go to the next task or dock at a certain base after serving. Therefore, we have designed a customized coding structure inspired by the investigation of priority encoding methods in logistics networks. Based on the symbols defined in section “Symbol description”,$\left| K \right|$represents the total number of tugboats and$\left| I \right|$represents the total number of tasks. Randomly generate a row matrix${v_{\left| K \right| \times \left| I \right|}}$of 1 row$\left| K \right| \times \left| I \right|$containing integers from 1 to$\left| K \right| \times \left| I \right|$, known as the priority matrix, and add a number after each priority, which is selected from the set $R=\{ 0,1,2, \ldots ,NL\}$. In addition, due to the distinction between tugboats and tasks, in individual decoding operations, it is necessary to form a row-column integer matrix${v_{\left| K \right|,\left| I \right|}}$. The specific operation method is to arrange the numbers of${v_{\left| K \right|,\left| I \right|}}$in order from left to right and then from top to bottom. Randomly generate a row-column integer matrix ${v_{ki}}$ containing the values from 1 to $\left| K \right| \times \left| I \right|$, which is the priority matrix, and add a number after each priority, which is selected from set$R=\{ 0,1,2, \ldots ,NL\}$.

If$\left| K \right|$is 3,$\left| I \right|$is 5, and$NL$is 2, then the feasible priority encoding schemes${v_{\left| K \right| \times \left| I \right|}}$and${v_{\left| K \right|,\left| I \right|}}$ can be represented as the ones shown in Tables 2 and 3, respectively. More information regarding the decoding operations (i.e., how the information presented in Tables 2 and 3 can be translated into a solution for the decision problem studied herein) is provided in section “Individual decoding operation” of the manuscript.

**Table 2:** A feasible priority encoding (form 1).

1	2	3	4	5	6	7	8	9	10	11	12	13	14	15
15(0)	1(2)	5(1)	14(2)	4(1)	7(2)	13(2)	11(0)	8(1)	10(1)	3(1)	12(2)	9(0)	2(2)	6(1)

**Table 3:** A feasible priority encoding (form 2).

	Task (docking base)				
Tugboat	1	2	3	4	5
1	15(0)	1(2)	5(1)	14(2)	4(1)
2	7(2)	13(2)	11(0)	8(1)	10(1)
3	3(1)	12(2)	9(0)	2(2)	6(1)

#### Individual decoding operation

This operation continues to use the symbolic meaning described in section “Symbol description” and proceeds as follows:

**Step 1**: Determine the input parameters, including${D_i}$,${P_k}$,${m_i}$,${Q_i}$, *N*, and priority${v_{ki}}$,$k \in K$,$i \in I$. Determine the output variables, including${x^{\prime}_{ki}}$.

**Step 2**: Determine the selection of 0 or 1 for the matrix${x^{\prime}_{ki}}$based on the inequality relationship in formulas (12), (17), and (18) and the priority relationship in the direction of the priority column${v_{ki}}$. In addition, because formula (18) contains two formulas, one of them is selected as the matching constraint with a 50% probability during this step of operation.

For example, continuing to use the data from Table 3, add the values for${m_i}$,${Q_i}$,${D_i}$,${P_k}$as shown in the first to third rows and second columns of Table 4. In addition, assuming *N* is 0.5, according to formula (12), the value range of$\sum\limits_{{i \in I}} {{{x^{\prime}}_{ki}}}$is between 1.333 and 6.666, indicating that the number of tugboats serving the task ranges from 2 to 6. Finally, obtain a 0–1 matrix from the fourth to sixth rows and the third to seventh columns in Table 4, which is the matrix${x^{\prime}_{ki}}$. For the fourth to sixth rows of the third column, if it is logical to choose the numbers 15, 7, and 3 in the second column of Table 3, then the position where the maximum number 15 is located is 1, and the rest are 0. The numbers in the fourth to sixth rows of the third column can be represented as [1,0,0]^T^. The same process can be applied to the other tasks.

**Table 4:** Matching scheme for tugboats.

Task required tugboat horsepower		350	400	400	350	400
Number of tugboats required for the specified horsepower of the task		0	2	2	0	2
The demand for tugboats		1	2	2	1	2
The horsepower of a tugboat	350	1	0	0	1	0
	450	0	1	1	0	1
	450	0	1	1	0	1

**Step 3**: Calculate the start time by inputting parameters, including${B_{kl}}$,$c_{{li}}^{1}$,$c_{{li}}^{2}$,$c_{{ij}}^{3}$, formula $\frac{{(\alpha - \lambda )w_{i}^{d}+(1 - \alpha )w_{i}^{c}}}{{1 - \lambda }}$, and priority${v_{ki}}$(horizontal direction):

Firstly, use Eq. (52) to calculate the time from a certain tugboat’s base to the first task:

52 $$s_{{ki}}^{0}=\hbox{max} \left\{ {{A_i},{{c_{{li}}^{1}} \mathord{\left/ {\vphantom {{c_{{li}}^{1}} {vc}}} \right. \kern-0pt} {vc}}} \right\}$$

Where *i* represents the task with the highest horizontal priority under the selected tugboat matching scheme, and *l* represents the initial docking base of tugboat *k*.

For subsequent tasks, Eq. (53) can be used as follows:

53 $$s_{{kj}}^{{n+1}}=\left\{ \begin{gathered} \hbox{max} \left\{ {{A_j},s_{{ki}}^{n}+{{c_{{ij}}^{3}} \mathord{\left/ {\vphantom {{c_{{ij}}^{3}} {vc}}} \right. \kern-0pt} {vc}}+\frac{{(\alpha - \lambda )w_{j}^{d}+(1 - \alpha )w_{j}^{c}}}{{1 - \lambda }}} \right\},\quad \quad \quad \;l=0 \hfill \\ \hbox{max} \left\{ {{A_j},s_{{ki}}^{n}+{{c_{{li}}^{2}} \mathord{\left/ {\vphantom {{c_{{li}}^{2}} {vc}}} \right. \kern-0pt} {vc}}+{{c_{{lj}}^{1}} \mathord{\left/ {\vphantom {{c_{{lj}}^{1}} {vc}}} \right. \kern-0pt} {vc}}+\frac{{(\alpha - \lambda )w_{j}^{d}+(1 - \alpha )w_{j}^{c}}}{{1 - \lambda }}} \right\},\;l \ne 0 \hfill \\ \end{gathered} \right.$$

Where *n*represents the first scheduled arrangement of preceding task *n*,$n+1$represents the current task arrangement,*j*represents the task corresponding to the second highest priority after task *i*, and *l*represents the docking base corresponding to the priority of task *i*.

For example, based on the data in Tables 3 and 4,$vc$is 5,$\alpha$is 0.9,$\lambda$is 0.1, and

$$c_{{li}}^{1} = c_{{li}}^{2} \left( {\begin{array}{*{20}c} 0 & {17} & {15} & {16} & {15} & {12} \\ 0 & {15} & {12} & {17} & {15} & {13} \\ \end{array} } \right)\;B_{{kl}} = \left( {\begin{array}{*{20}c} 0 & 0 & 1 \\ 0 & 1 & 0 \\ 0 & 0 & 1 \\ \end{array} } \right)\;c_{{ij}}^{3} = \left( {\begin{array}{*{20}c} {17} & {13} & {15} & {15} & {12} \\ {15} & {12} & {17} & {15} & {13} \\ {13} & {15} & {17} & {15} & {12} \\ {12} & {17} & {15} & {15} & {13} \\ {17} & {15} & {13} & {15} & {12} \\ \end{array} } \right)$$

$$w_{i}^{c} = \left( {\begin{array}{*{20}c} 7 & 7 & 7 & 7 \\ \end{array} } \right),\;w_{i}^{d} = \left( {\begin{array}{*{20}c} 8 & 8 & 8 & 8 \\ \end{array} } \right)$$

For tugboat 1, due to the selection of tasks 1 and 4, and based on their priorities 15 and 14, the order in which tugboat 1 executes tasks is 1, 4, and the following relationships will apply:

$$s_{{11}}^{0}=\hbox{max} \left\{ {{A_1},{{c_{{21}}^{1}} \mathord{\left/ {\vphantom {{c_{{21}}^{1}} {vc}}} \right. \kern-0pt} {vc}}} \right\}=\hbox{max} \left\{ {20,{{17} \mathord{\left/ {\vphantom {{17} 5}} \right. \kern-0pt} 5}} \right\}=20$$,

$$\begin{aligned} s_{{14}}^{1}&=\hbox{max} \left\{ {{A_4},s_{{11}}^{0}+{{c_{{14}}^{3}} \mathord{\left/ {\vphantom {{c_{{14}}^{3}} {vc}}} \right. \kern-0pt} {vc}}+\frac{{(\alpha - \lambda )w_{4}^{d}+(1 - \alpha )w_{4}^{c}}}{{1 - \lambda }}} \right\} \hfill \\ &=\hbox{max} \left\{ {40,20+{{15} \mathord{\left/ {\vphantom {{15} 5}} \right. \kern-0pt} 5}+7.888} \right\}=40 \hfill \\ \end{aligned}$$.

For tugboat 2, due to the selection of tasks 2, 3, and 5, based on their priorities 13, 11, and 10, the order in which tugboat 2 executes tasks is 2, 3, and 5

$$s_{{22}}^{0}=\hbox{max} \left\{ {{A_2},{{c_{{12}}^{1}} \mathord{\left/ {\vphantom {{c_{{12}}^{1}} {vc}}} \right. \kern-0pt} {vc}}} \right\}=\hbox{max} \left\{ {1,{{15} \mathord{\left/ {\vphantom {{15} 5}} \right. \kern-0pt} 5}} \right\}=3$$,

$$\begin{aligned} s_{{23}}^{2}&=\hbox{max} \left\{ {{A_3},s_{{22}}^{0}+{{c_{{22}}^{2}} \mathord{\left/ {\vphantom {{c_{{22}}^{2}} {vc}}} \right. \kern-0pt} {vc}}+{{c_{{23}}^{1}} \mathord{\left/ {\vphantom {{c_{{23}}^{1}} {vc}}} \right. \kern-0pt} {vc}}+\frac{{(\alpha - \lambda )w_{3}^{d}+(1 - \alpha )w_{3}^{c}}}{{1 - \lambda }}} \right\} \hfill \\ &=\hbox{max} \left\{ {30,3+{{12} \mathord{\left/ {\vphantom {{12} 5}} \right. \kern-0pt} 5}+{{17} \mathord{\left/ {\vphantom {{17} 5}} \right. \kern-0pt} 5}+7.888} \right\}=30 \hfill \\ \end{aligned}$$,

$$\begin{aligned} s_{{25}}^{3}&=\hbox{max} \left\{ {{A_5},s_{{23}}^{2}+{{c_{{35}}^{3}} \mathord{\left/ {\vphantom {{c_{{35}}^{3}} {vc}}} \right. \kern-0pt} {vc}}+\frac{{(\alpha - \lambda )w_{5}^{d}+(1 - \alpha )w_{5}^{c}}}{{1 - \lambda }}} \right\} \hfill \\ &=\hbox{max} \left\{ {45,30+{{12} \mathord{\left/ {\vphantom {{12} 5}} \right. \kern-0pt} 5}+7.888} \right\}=45 \hfill \\ \end{aligned}$$.

### Features of the seagull optimization algorithm

The standard seagull optimization algorithm obtains the optimal flight position by simulating the migration and attack behavior of seagulls within a specific search area. Seagulls are a globally distributed group of seabirds that migrate between different regions in search of food as the seasons change. The predation process of seagulls mainly includes the migration stage and the attack stage. During the migration process, when all seagulls fly towards the position with the most abundant food, individuals maintain independence according to a certain flight pattern to avoid positional collisions. Meanwhile, to obtain more food, seagulls usually engage in spiral-flight attacks on the prey.

The standard seagull optimization algorithm is only suitable for optimizing in a continuous solution space. However, the solution space of the problem studied in this article is discrete, and the standard seagull optimization algorithm needs to be discretized to make it suitable for solving tugboat scheduling problems. For metaheuristic algorithms that solve continuous problems, it is necessary to redefine the position update strategy in the algorithm to adapt to discrete encoding problems. In the cuckoo bird optimization algorithm, Zheng et al.^50^ used a path reconnection strategy instead of the Lévy flight to generate new paths through swap and insert methods. Teymourian et al.^51^ used the 2-opt method to replace the Lévy flight. Alssager and Othman^52^ used local search algorithms to search in large neighborhoods instead of the Lévy flight but did not specify which local search algorithms were used. In addition, Gezici and Livatyali^53^ proposed a corresponding metaheuristic optimization algorithm for discrete optimization problems based on a population-based metaheuristic optimization algorithm for continuous optimization problems. The algorithm modified the way new solutions were produced, and its application was demonstrated using the CEC2019 test function and a three-dimensional packing problem (3D-BPP) dataset with 320 samples. After studying the discretization process of various continuous algorithms, this paper adopted a discrete update strategy suitable for the priority encoding of seagull optimization algorithms.

#### Migration operations

Seagulls may not directly search for the neighborhood of the optimal solution during their migration process, but they tend to explore good-quality solutions around them. In order to explore good-quality solutions, facilitate the global evolution direction of the population, and ensure the flight independence of seagulls, this study deployed the strategy of inserting and reversing the best obtained path scheme with a 50% probability of developing the neighborhood of the best solution identified, so that seagulls can migrate towards the neighborhood of the best position. Taking the data in Table 2 as an example, the insert operation (see Table 5) randomly selects two nodes *i* and *j* in the best path scheme identified, inserts node *j* next to the position of node *i*, and forms a new path. The reverse operation (see Table 6) aims to randomly select two nodes *i* and *j* in the best path scheme identified, invert all nodes between node *i* and node *j*, and form a new path.

**Table 5:** Insert operation.

Before updating:														
				*i*				*j*						
15(0)	1(2)	5(1)	14(2)	4(1)	7(2)	13(2)	11(0)	8(1)	10(1)	3(1)	12(2)	9(0)	2(2)	6(1)

After update:														
				*i*	*j*									
15(0)	1(2)	5(1)	14(2)	4(1)	8(1)	7(2)	13(2)	11(0)	10(1)	3(1)	12(2)	9(0)	2(2)	6(1)

**Table 6:** Reverse operation.

Before updating:														
					*i*				*j*					
15(0)	1(2)	5(1)	14(2)	4(1)	7(2)	13(2)	11(0)	8(1)	10(1)	3(1)	12(2)	9(0)	2(2)	6(1)

After update:														
					j				i					
15(0)	1(2)	5(1)	14(2)	4(1)	10(1)	8(1)	11(0)	13(2)	7(2)	3(1)	12(2)	9(0)	2(2)	6(1)

#### Attack operations

When seagulls attack their prey, the method of using a spiral flight is relatively common. To expand the local search range and improve the ability to discover new paths, based on the final migration location of seagulls, swap and 3-opt operations are performed on the current path scheme to replicate seagull attack methods. The swap operation (see Table 7) is conducted by randomly selecting two nodes *i* and *j*in the selected path scheme and exchanging the positions of node *i* and node *j*to form a new path. The 3-opt operation (see Table 8) is performed by randomly selecting three nodes *i*, *j*, and *k*from the selected path scheme and exchanging the positions of the three nodes to form a new path.

**Table 7:** Swap operation.

Before updating:														
				*i*				*j*						
15(0)	1(2)	5(1)	14(2)	4(1)	7(2)	13(2)	11(0)	8(1)	10(1)	3(1)	12(2)	9(0)	2(2)	6(1)

After update:														
				*j*				*i*						
15(0)	1(2)	5(1)	14(2)	8(1)	7(2)	13(2)	11(0)	4(1)	10(1)	3(1)	12(2)	9(0)	2(2)	6(1)

**Table 8:** 3-opt operation.

Before updating:														
				*i*				*j*				*k*		
15(0)	1(2)	5(1)	14(2)	4(1)	7(2)	13(2)	11(0)	8(1)	10(1)	3(1)	12(2)	9(0)	2(2)	6(1)

After update:														
				*j*				k				*i*		
15(0)	1(2)	5(1)	14(2)	9(0)	7(2)	13(2)	11(0)	9(0)	10(1)	3(1)	12(2)	4(1)	2(2)	6(1)

### Fitness function and selection operations

Due to the need for multiple tugboats to be used simultaneously in the task, the encoding method mentioned above may cause start time conflicts after decoding. Generally speaking, there are two types of conflicts:

1. Conflict situation 1: For task *i*, check whether$s_{{ki}}^{n}$is within the range of the task time window$\left[ {{A_i},{A_i}+{\tau _i}} \right]$. Moreover, for task$i \in I^{\prime}$, check whether$s_{{ki}}^{n}+\frac{{(\alpha - \lambda )w_{i}^{d}+(1 - \alpha )w_{i}^{c}}}{{1 - \lambda }}$is within the range of task time window$\left[ {{A_i},{A_i}+{\tau _i}} \right]$. If not, add the corresponding penalty function to the objective function Eq. (49) as follows:
  54 $$\hbox{max} \;\psi {\lambda _0}+(1 - \psi )\left[ {{\theta _1}{\mu _1}(v)+{\theta _2}{\mu _2}(v)+{\theta _3}{\mu _3}(v)} \right] - {F_1} \times \sum\limits_{{k \in K}} {\sum\limits_{{i \in I}} {\left( {s_{{ki}}^{n} - {A_i} - {\tau _i}} \right)} }$$
  Where${F_1}$is the penalty value.
2. Conflict situation 2: When each tugboat is performing the same task, it must be carried out simultaneously at a certain period. It is not allowed for one tugboat to complete the task and start the next task before another tugboat starts the task that was scheduled for simultaneous execution.
  The minimum and maximum start times for a task are$s_{i}^{{\hbox{min} }}=\hbox{min} \left\{ {s_{{ki}}^{n}} \right\}$and$s_{i}^{{\hbox{max} }}=\hbox{max} \left\{ {s_{{ki}}^{n}} \right\}$, respectively. The tugboat *k* that performs task *i*, for all$j \in I\backslash \left\{ i \right\}$, if both$s_{{kj}}^{n}<s_{i}^{{\hbox{max} }}$and$s_{{kj}}^{n}+{y_{kj}}>s_{i}^{{\hbox{min} }}$are met, then task *j*, intersects with task *i*. Firstly, set an empty matrix${\Delta _{kij}}$for tugboat *k* that performs task *i*. For each *j*, if$s_{{kj}}^{n}<s_{i}^{{\hbox{max} }}$and$s_{{kj}}^{n}+{y_{kj}}>s_{i}^{{\hbox{min} }}$are not met, then${\Delta _{kij}}$ = 0. For tugboat *k*that does not perform task *i*, then${\Delta _{kij}}$ = 0 for each *i*and *j*. Otherwise, proceed to the next step:
  If$s_{{kj}}^{n}<s_{i}^{{\hbox{min} }}$and$s_{{kj}}^{n}+{y_{kj}} \leqslant s_{i}^{{\hbox{max} }}$, then${\Delta _{kij}}=s_{{kj}}^{n}+{y_{kj}} - s_{i}^{{\hbox{min} }}$;
  If$s_{{kj}}^{n} \geqslant s_{i}^{{\hbox{min} }}$and$s_{{kj}}^{n}+{y_{kj}}>s_{i}^{{\hbox{max} }}$, then${\Delta _{kij}}=s_{i}^{{\hbox{max} }} - s_{{kj}}^{n}$;
  If$s_{{kj}}^{n}<s_{i}^{{\hbox{min} }}$and$s_{{kj}}^{n}+{y_{kj}}>s_{i}^{{\hbox{max} }}$, then${\Delta _{kij}}=s_{i}^{{\hbox{max} }} - s_{i}^{{\hbox{min} }}$.
  The corresponding penalty function needs to be added to Eq. (54) as follows:
  55 $$\begin{gathered} \hbox{max} \;\psi {\lambda _0}+(1 - \psi )\left[ {{\theta _1}{\mu _1}(v)+{\theta _2}{\mu _2}(v)+{\theta _3}{\mu _3}(v)} \right] - {F_1} \times \sum\limits_{{k \in K}} {\sum\limits_{{i \in I}} {\left( {s_{{ki}}^{n} - {A_i} - {\tau _i}} \right)} } \hfill \\ \quad \quad - {F_2} \times \sum\limits_{{k \in K}} {\sum\limits_{{i \in I}} {\sum\limits_{{j \in I}} {{\Delta _{kij}}} } } \hfill \\ \end{gathered}$$
  Where${F_2}$is the penalty value.
  Therefore, the fitness value to be used for quantifying the quality of solutions in the seagull optimization algorithm can be expressed as follows:
  56 $$\begin{gathered} objective=\psi {\lambda _0}+(1 - \psi )\left[ {{\theta _1}{\mu _1}(v)+{\theta _2}{\mu _2}(v)+{\theta _3}{\mu _3}(v)} \right] - {F_1} \times \sum\limits_{{k \in K}} {\sum\limits_{{i \in I}} {\left( {s_{{ki}}^{n} - {A_i} - {\tau _i}} \right)} } \hfill \\ \quad \quad \quad \quad - {F_2} \times \sum\limits_{{k \in K}} {\sum\limits_{{i \in I}} {\sum\limits_{{j \in I}} {{\Delta _{kij}}} } } \hfill \\ \end{gathered}$$
  Due to the existence of penalty values, Eq. (56) may produce negative values. Therefore, it is necessary to use Eq. (57) to turn all negative values into positive values. Let the current population set be$t \in T$, and$objectiv{e_t}$be the objective value of the individual *t*. Then, the fitness function can be represented as follows:
  57 $$fitnes{s_t}=\mathop {\hbox{max} }\limits_{{t \in T}} \left\{ {\left| {objcetiv{e_t}} \right|} \right\}+objcetiv{e_t}$$
  The roulette wheel selection method was adopted in this study as a selection mechanism. Assuming the population size is$pop\_szie$, due to the crossover operation applied to the population in this algorithm, the population size becomes twice the original population size, i.e., $2 \times pop\_szie$. During the selection operation, the roulette wheel needs to be rotated $pop\_szie$ times. The individuals identified by the roulette wheel selection mechanism will be designated as seagulls that will be moved to the next iteration and continue the search process.

### Crossover and mutation operations

By taking two different nodes *i* and *j*from two different individuals (parents), the middle part is exchanged by the developed crossover operator to replace the mapping relationship of the exchange part for scenarios where there are no exchanges but duplicate numbers with the exchange part. As shown in Table 9, after exchanging the parts between two random nodes, the changes are made through the mapping relationship of 1$\leftrightarrow$4$\leftrightarrow$13, 9$\leftrightarrow$12$\leftrightarrow$3, and 2$\leftrightarrow$10, as shown by the bold and underlined numbers in Table 9. After the crossover operation, the mutation operation is performed as well. The mutation operation is mainly aimed at changing the docking base, as shown in Table 10. The developed mutation operator randomly selects node *i* and then changes the docking base (as shown in bold underlined numbers in Table 10).

**Table 9:** Cross operation.

Before updating:														
Parent 1			*i*								*j*			
15(0)	1(2)	5(1)	14(2)	4(1)	7(2)	13(2)	11(0)	8(1)	10(1)	3(1)	12(2)	9(0)	2(2)	6(1)

Parent 2			*i*								*j*			
10(1)	5(1)	15(0)	6(1)	1(2)	11(0)	4(1)	8(2)	14(2)	2(0)	12(2)	9(1)	13(2)	3(1)	7(0)

Before updating:														
Descendant 1			*i*								*j*			
15(0)	**13**(2)	5(1)	6(1)	1(2)	11(0)	4(1)	8(2)	14(2)	2(0)	12(2)	9(1)	**3**(0)	**10**(2)	6(1)

Before updating:														
Descendant 2			*i*								*j*			
**2**(1)	5(1)	15(0)	14(2)	4(1)	7(2)	13(2)	11(0)	8(1)	10(1)	3(1)	12(2)	**1**(2)	**9**(1)	7(0)

**Table 10:** Mutation operation.

Before updating:														
								*i*						
15(0)	1(2)	5(1)	14(2)	4(1)	7(2)	13(2)	11(0)	8(1)	10(1)	3(1)	12(2)	9(0)	2(2)	6(1)

After update:														
								*i*						
15(0)	1(2)	5(1)	14(2)	4(1)	7(2)	13(2)	11(0)	8(**2**)	10(1)	3(1)	12(2)	9(0)	2(2)	6(1)

## Numerical experiments

### Input data generation

To evaluate the performance of the proposed Seagull Optimization Algorithm based on Priority Encoding and Genetic Operators (SOAPG), CPLEX 12.8, traditional genetic algorithm (GA), discrete seagull optimization algorithm (SOA), and simulated annealing algorithm (SA) were considered during the computational experiments as well. For fair comparison, the encoding of GA, SOA, and SA was performed following the encoding method proposed in this research. Furthermore, the crossover and mutation operations used within GA were similar to the ones described in section “Fitness function and selection operations”. The migration and attack operations used within SOA were similar to the ones described in section “Features of the seagull optimization algorithm”. The SA operations for generating new solutions were similar to the ones described in section “Migration operations”. The codes for SOAPG, GA, SOA, and SA were compiled in MATLAB 7.0. The running environment for software MATLAB 7.0 and CPLEX was composed of an Intel (R) Core (TM) i7 2.70 GHz processor, 4GB memory, and a Windows 10 (64bit) laptop operating system. This study randomly generated 45 problem instances of different scales based on Tables 11, 12, 13, 14, 15 and 16. Among them, Tables 11 and 12, and 14 were obtained based on data from the literature^5^. Table 13 was provided by internal resources of the Guangzhou Port, and Table 15 was obtained based on the information presented in the “Guangdong Province Port Ship Berthing and Departure and Pilot or Transfer Tug Ship Allocation Standards”.

**Table 11:** Number of tugboats with different horsepower capabilities in various port areas of Guangzhou.

Port area	Horsepower type				
	1600HP	3000HP	4000HP	5000HP	Above 6000HP
Huangpu	1	4	0	0	0
Xinsha	0	3	1	2	0
Xiaohu	0	0	3	1	0
Nansha	0	0	5	2	3
Total	1	7	9	5	3

**Table 12:** Fuel consumption per unit of time for each tugboat type.

${P_k}$	$Co$(kg/min)	$\gamma$(kg/min)
1600HP	6.33	2.45
3000HP	6.67	2.51
4000HP	7.50	2.67
5000HP	10.83	2.92
6000HP	11.25	3.67
6800HP	11.50	4.33
6900HP	11.67	4.33

**Table 13:** Overview of each channel of the Guangzhou Port’s seaway.

Number	Name	Length (km)	Average Speed (km/h)	Average sailing time (min)
1	Dahao Waterway	18.8	12.53	90
2	South Section of Lingding Waterway	28.7	11.48	150
3	North Section of Lingding Waterway	22	11.00	120
4	Chuanbi Waterway	11.9	7.93	90
5	Dahu Waterway	8.2	9.87	50
6	Nizhou Waterway	8.6	11.46	45
7	Lianhua Mountain West Waterway	7.8	10.40	45
8	Lianhua Mountain East Waterway	7.7	10.26	45
9	Xinsha Waterway	7.1	10.65	40

Generally speaking, the speed limit for tugboats in Guangzhou Port is 10 knots, while the maximum speed in other regions is 13 knots. From the nine average speeds in Table 13, it can be inferred that the average speed of tugboats in the Guangzhou Port on each sea route is 10.62 km/h, which meets the requirements. According to Table 13, the distance from the task to the base can be set between 7 km and 28 km, and the distance between tasks can be set between 2 km and 28 km.

**Table 14:** Tugboat configuration for berthing and unberthing operations (including mooring and unberthing buoys) under normal conditions and special water areas in Guangzhou Port.

Water area	Length of vessel (L)/m	Number of tugboats (nos.)	Requirements for tugboat horsepower
Usual conditions	80$\:\le\:$L<120	1	-
	120$\:\le\:$L<180	2	One tugboat with no less than 3000HP
	180$\:\le\:$L<230	2	Each tugboat’s horsepower is no less than 3000HP
	230$\:\le\:$L<270, D < 11	2	Each tugboat’s horsepower is no less than 4000HP
	230$\:\le\:$L<270, D$\:\ge\:$11	3	Two of the tugboats have no less than 4000HP each
	270$\:\le\:$L<390	3	Two of the tugboats have no less than 4000HP each
	390$\:\le\:$L	3	Two of the tugboats have no less than 5000HP each
Xiaohu Petrochemical’s internal archives, Gangfa, Nanwei, and Nansha Freight Terminals	L < 80	1	-
	80$\:\le\:$L<120	2	Each tugboat’s horsepower is no less than 1600HP
	120$\:\le\:$L<180	2	One tugboat with no less than 3000HP
	180$\:\le\:$L<230	2	Each tugboat’s horsepower is no less than 3000HP
	L$\:\ge\:$230	3	Two of the tugboats have no less than 4000HP each
Hongye Wharf	L < 80	1	-
	80$\:\le\:$L<150	2	One tugboat with no less than 3000HP
	150$\:\le\:$L<230	2(Berthing) 3(Unberthing)	Each tugboat’s horsepower is no less than 3000HP(Berthing) Two of the tugboats have no less than 3000HP each (Unberthing)
	L$\:\ge\:$230	3	Two of the tugboats have no less than 4000HP each

**Table 15:** Table of tugboat configuration for top flow, downstream turning anchor, and anchor turning operation under normal conditions in Guangzhou Port.

Water area	Tide-rode Length of vessel (L)/m	Downstream turning anchor Length of vessel (L)/m	Anchor turning Length of vessel (L)/m	Number of tugboats (nos.)
Water area between Ma Youshi and Humen Bridge	L$\:\ge\:$180	L$\:\ge\:$150	L$\:\ge\:$150	1
Water area between Humen Bridge and Dahaozhou	L$\:\ge\:$150	L$\:\ge\:$120	-	1
Water area between Humen Bridge and Nizhoutou	-	-	L$\:\ge\:$120	1
Water area between Nizhoutou and Dahaozhou	-	-	L$\:\ge\:$100	1
Water area between Huangpu Port and Inner Harbour	L$\:\ge\:$100	L$\:\ge\:$80	L$\:\ge\:$80	1

In addition, in practical operations, the total power method is also used, which means that the total power provided by the available number of tugs with a certain power multiplied by their respective horsepower should be greater than the required power. As shown in Table 14, when 270 ≤ L < 390, the total power of tugs serving the vessel needs to be greater than 2$\times$4000HP = 8000HP.

Overall, the maximum operating time for one tugboat was set to 15–45 min, for two tugboats it was set to 25–60 min, and for three tugboats it was assumed to comprise 30–75 min. When working with the available data for planning purposes, the port side generally knows in advance the vessel type and related operational information of the incoming vessels, so they can determine in advance the required configuration of tugboats for these vessels.

**Table 16:** Scale of problem instances (unit: units).

Example	Task	Tugboat	Base	Example	Task	Tugboat	Base
1	5	3	2	24	28	16	11
2	6	4	2	25	29	17	12
3	7	5	2	26	30	18	12
4	8	6	3	27	31	18	13
5	9	6	3	28	32	18	13
6	10	6	4	29	33	19	13
7	11	7	5	30	34	20	14
8	12	7	5	31	35	21	14
9	13	8	5	32	36	21	14
10	14	8	6	33	37	22	15
11	15	9	6	34	39	23	15
12	16	9	6	35	40	24	16
13	17	10	7	36	43	25	16
14	18	10	7	37	45	26	16
15	19	11	7	38	47	27	16
16	20	12	8	39	49	28	16
17	21	13	9	40	50	30	16
18	22	13	9	41	52	31	16
19	23	14	9	42	54	33	16
20	24	14	10	43	56	34	16
21	25	15	10	44	58	35	16
22	26	15	10	45	60	36	16
23	27	16	11				

### Evaluation of the candidate solution approaches

In order to facilitate the verification of the performance of the proposed SOAPG, objective 1 was adopted for all the four considered solution approaches, namely:

58 $$objective={f_1} - {F_1} \times \sum\limits_{{k \in K}} {\sum\limits_{{i \in I}} {\left( {s_{{ki}}^{n} - {A_i} - {\tau _i}} \right)} } - {F_2} \times \sum\limits_{{k \in K}} {\sum\limits_{{i \in I}} {\sum\limits_{{j \in I}} {{\Delta _{kij}}} } }$$

In addition, the fitness formula follows Eq. (57), and the objective value function of CPLEX follows Eq. (1). The ambiguity coefficients were assumed to be $\alpha$=$\beta$=$\lambda ^{\prime}$=0.5, ${F_1}$=10^5^,${F_2}$=10^6^. Furthermore, a set of parameter tuning experiments were conducted to set the appropriate values of the considered metaheuristic algorithms. In particular, based on the parameter tuning analysis, the crossover and mutation rates of SOAPG and GA were set to 60%~70% and 10%~15%, respectively. Each algorithm was run 20 times for each problem instance, and the results are reported in Tables 17 and 18 (bold numbers represent the optimal solution, and those marked with “*” represent feasible solutions). The sentence “Did not converge” indicates that CPLEX identified a feasible solution but could not find the optimal solution within the time limit imposed (which is 9,000 s).

**Table 17:** Objective function values and CPU times of the considered solution approaches (CPLEX, SOAPG, and GA).

Example(Population size, Iterations’ number)	CPLEX		SOAPG			GA		
	Objective value	CPU time (seconds)	Objective value	Average value	CPU time (seconds)	Objective value	Average value	CPU time (seconds)
1(50,100)	**55**,**945.80**	3.38	**55**,**945.80**	56,657.20	62.51	**55**,**945.80**	57,547.38	56.68
2(50,100)	**56**,**040.66**	4.04	**56**,**040.66**	58,637.38	72.01	**56**,**040.66**	59,543.01	63.52
3(50,100)	**56**,**135.52**	4.71	**56**,**135.52**	58,836.43	78.36	**56**,**135.52**	59,638.49	69.57
4(50,100)	**56**,**230.38**	5.37	**56**,**230.38**	58,935.65	86.57	**56**,**230.38**	59,865.64	75.22
5(50,100)	**56**,**325.24**	6.03	**56**,**325.24**	59,465.33	90.62	**56**,**325.24**	60,475.33	81.35
6(100,150)	**56**,**420.10**	6.70	**56**,**420.10**	60,254.32	96.27	**56**,**420.10**	61,354.24	87.24
7(100,150)	**59**,**919.15**	15.59	**59**,**919.15**	61,349.02	102.15	**59**,**919.15**	62,267.19	94.35
8(100,150)	**63**,**418.20**	24.48	**63**,**418.20**	64,235.34	115.33	**63**,**418.20**	65,258.69	106.59
9(100,150)	**66**,**917.25**	33.37	**66**,**917.25**	67,536.92	118.67	**66**,**917.25**	68,741.82	109.71
10(100,150)	**70**,**416.30**	42.27	**70**,**416.30**	72,625.35	131.06	71,057.12	73,159.53	120.28
11(200,300)	**73**,**915.35**	51.16	**73**,**915.35**	75,269.56	154.25	74,613.02	76,752.26	137.45
12(200,300)	**77**,**414.40**	60.05	**77**,**414.40**	79,512.07	173.64	78,251.33	81,236.60	159.20
13(200,300)	**80**,**913.45**	68.94	**80**,**913.45**	82,347.36	195.02	81,641.37	83,068.57	180.56
14(200,300)	**84**,**412.50**	77.83	**84**,**412.50**	86,542.35	216.84	85,361.22	87,940.68	203.81
15(200,300)	**87**,**911.55**	86.72	**87**,**911.55**	89,046.29	235.35	88,146.54	90,248.51	224.23
16(250,350)	**91**,**410.60**	95.62	**91**,**410.60**	92,345.32	258.30	92,236.07	93,753.68	245.55
17(250,350)	**93**,**034.38**	365.09	**93**,**034.38**	95,246.37	271.54	95,032.71	96,657.42	259.48
18(250,350)	**94**,**658.16**	634.56	**94**,**658.16**	96,145.13	302.23	95,346.98	97,862.69	284.64
19(250,350)	**96**,**281.94**	904.03	**96**,**281.94**	98,254.71	326.09	97,952.63	99,861.65	309.53
20(250,350)	**97**,**905.72**	1173.50	**97**,**905.72**	99,694.65	350.47	98,657.37	100,591.88	336.52
21(300,400)	**99**,**529.50**	1442.92	**99**,**529.50**	100,543.52	362.64	100,475.24	101,947.62	354.85
22(300,400)	**101**,**153.28**	1712.44	101,243.63	103,294.95	396.22	102,574.68	104,133.86	371.39
23(300,400)	**102**,**777.06**	1981.91	102,951.26	103,876.56	420.43	103,536.09	104,472.48	406.08
24(300,400)	**104**,**400.84**	2251.38	104.519.86	105,452.68	463.24	104,946.27	106,865.23	432.67
25(300,400)	**106**,**024.62**	2520.85	106,237.18	108,056.26	493.57	106,937.36	109,342.06	470.89
26(400,500)	**107**,**648.40**	2790.32	107,836.17	108,951.34	512.65	108,365.33	109,842.37	498.21
27(400,500)	**109**,**911.31**	2815.24	110,563.32	111,645.55	531.03	111,124.65	112,456.83	519.26
28(400,500)	**112**,**174.22**	2863.37	112,537.34	113,098.37	556.45	113,249.74	114,254.47	535.27
29(400,500)	**114**,**437.13**	2812.25	114,836.27	115,297.22	573.01	115,674.54	116,783.49	561.85
30(400,500)	**116**,**700.04**	2954.13	116,923.01	118,635.42	589.62	118,357.79	119,864.66	576.83
31(450,600)	**120**,**304.05**	2982.51	120,476.37	121,421.89	603.54	121,159.59	122,498.22	593.20
32(450,600)	**121**,**225.86**	5632.79	121,563.51	122,489.08	621.30	122,258.40	123,547.82	611.16
33(450,600)	123,488.77*(Heuristic still looking.)	9000+	122,826.36	123,743.90	652.27	123,432.74	124,574.69	639.18
34(450,600)	125,751.68*(Heuristic still looking.)	9000+	124,237.25	125,694.72	669.26	126,327.07	127,803.40	648.49
35(450,600)	128,014.64*(Heuristic still looking.)	9000+	126,357,22	127,364.04	685.33	127,687.42	128,259.82	672.30
36(500,700)	–	–	127,261.41	128,261.95	706.52	128,237.91	129,382.64	686.29
37(500,700)	–	–	127,534.08	128,638.68	726.11	128,864.25	129,765.86	708.43
38(500,700)	–	–	128,162.30	129,236.84	735.02	129,421.57	131,589.31	725.29
39(500,700)	–	–	128,637.65	129,778.31	756.98	129,632.04	131,625.09	735.95
40(500,700)	–	–	129,251.82	131,591.69	768.32	130,234.01	132,147.20	759.37
41(600,900)	–	–	129,735.21	132,547.33	784.32	130.951.34	132,628.55	764.65
42(600,900)	–	–	130,235.01	133,698.79	802.53	131,654.21	134,320.03	783.09
43(600,900)	–	–	131,952.76	133,869.26	815.43	132,684.18	134,586.67	798.43
44(600,900)	–	–	132,697.32	134,694.35	839.81	133,547.65	135,049.40	820.85
45(600,900)	–	–	133,246.65	135,489.56	854.69	134,658.49	136,852.74	842.67

**Table 18:** Objective function values and CPU times of the considered solution approaches (SOA and SA).

Example(population size, Iterations’ number)	SOA			SA		
	Objective value	Average value	CPU time (seconds)	Objective value	Average value	CPU time (seconds)
1(50,100)	**55**,**945.80**	57,726.35	59.24	**55**,**945.80**	57,542.33	60.57
2(50,100)	**56**,**040.66**	57,861.42	61.58	**56**,**040.66**	57,489.30	62.35
3(50,100)	**56**,**135.52**	58,365.86	67.23	**56**,**135.52**	58,567.41	68.47
4(50,100)	**56**,**230.38**	58,751.93	73.55	**56**,**230.38**	58,698.75	72.57
5(50,100)	**56**,**325.24**	59,537.23	78.29	**56**,**325.24**	59,547.20	79.58
6(100,150)	**56**,**420.10**	59,943.07	85.20	**56**,**420.10**	59,846.21	87.25
7(100,150)	**59**,**919.15**	63,679.45	92.17	**59**,**919.15**	62,756.05	94.33
8(100,150)	**63**,**418.20**	65,307.59	101.34	**63**,**418.20**	65,789.62	103.58
9(100,150)	**66**,**917.25**	68,247.35	107.46	**66**,**917.25**	68,574.10	108.37
10(100,150)	**70**,**416.30**	72,354.21	116.74	73,687.38	75,123.60	117.22
11(200,300)	74,547.09	76,523.46	131.08	74,869.32	75,741.59	132.62
12(200,300)	78,258.60	80,359.31	156.92	78,569.27	79,482.39	157.54
13(200,300)	81,569.54	83,497.25	177.54	81,569.68	83,498.62	177.84
14(200,300)	85,154.62	86,764.59	191.95	85,476.57	87,258.93	190.35
15(200,300)	87,637.52	89,576.48	219.51	87,739.51	89,328.43	218.26
16(250,350)	92,048.75	93,597.56	236.04	92,468.53	93,663.42	237.08
17(250,350)	94.967.43	95,267.24	248.16	94,246.08	96,272.51	251.49
18(250,350)	95,241.03	96,513.76	267.86	95,547.21	97,635.55	270.30
19(250,350)	97,537.89	98,421.32	296.49	97,637.42	99,884.21	299.57
20(250,350)	98,468.37	99.637.21	319.43	98,787.37	100,699.30	322.01
21(300,400)	100,257.16	101,576.35	341.58	100,384.65	102,587.47	345.24
22(300,400)	101,864.45	103,468.73	359.24	101,657,52	103,767.66	361.35
23(300,400)	103,423.72	105,374.68	387.41	103,423.80	105,154.35	388.75
24(300,400)	104,819.47	105,954.27	426.33	104,523.14	105,928.64	428.31
25(300,400)	106,754.80	108,469.30	462.69	106,639.25	108,637.20	464.78
26(400,500)	109,057.15	110,863.47	486.26	109,394.33	110,852.94	491.95
27(400,500)	111,967.53	112,637.20	508.52	111,638.32	112,264.21	512.22
28(400,500)	113,067.64	115,563.19	524.61	113,207.42	115,885.43	529.25
29(400,500)	116,324.99	118,465.17	552.03	116,389.76	118,525.56	558.66
30(400,500)	119,384.20	120,742.95	563.80	119,627.20	120,541.13	569.24
31(450,600)	120,932.26	121,649.39	589.58	121,159.34	123,288.95	595.19
32(450,600)	122,132.68	123,847.46	603.39	122,415.66	124,963.47	608.49
33(450,600)	123,256.53	125,498.25	625.48	123,854.57	125,227.30	628.65
34(450,600)	126,568.32	127,864.59	636.05	126,837.62	127,626.08	640.09
35(450,600)	127,820.60	128,657.34	659.83	128,687.04	130,212,32	662.43
36(500,700)	129,104.37	130,378.92	670.54	129,563.18	131,545.17	672.62
37(500,700)	129,634.90	132,156.36	695.71	130,691.36	132,682.66	701.31
38(500,700)	131,196.84	133,468.37	716.43	131,257.86	133,236.52	715.60
39(500,700)	131,541.22	133,835.96	723.28	131,630.54	133,894.54	726.42
40(500,700)	131,935.70	133,579.63	742.60	132,045.75	134,427.70	748.05
41(600,900)	132,309.08	134,549.05	754.26	132,952.34	134,853.64	756.35
42(600,900)	133,865.24	134,687.54	772.13	133,985.47	135,527.41	786.20
43(600,900)	134,286.12	136,579.58	785.02	135,204.55	137,152.86	793.84
44(600,900)	134,683.07	136,289.67	811.24	135,810.21	137,656.28	816.32
45(600,900)	136,335.68	138,479.53	836.37	137,023.68	139,726.01	839.33

As shown in Table 17, for problem instances 1–16, the objective values obtained by CPLEX and SOAPG are the same, but the solving time used by SOAPG is fairly longer than that used by CPLEX, indicating that for small-scale examples, CPLEX has high efficiency. For problem instances 17–21, although the objective values obtained by CPLEX and SOAPG are also the same, the solving time used by CPLEX is gradually much longer than that of SOAPG, indicating that for small to medium-scale examples, the efficiency of SOAPG begins to improve. For problem instances 22–32, although the objective values obtained by CPLEX are still optimal, CPLEX required too much time to discover these optimal solutions. Although SOAPG could not find the optimal objective values for problem instances 22–32, the obtained objective values are not much larger than the optimal ones and can be viewed as acceptable. Moreover, the solving time required by SOAPG is much shorter than that required by CPLEX. This indicates that for medium to large-scale examples, the accuracy of SOAPG may slightly decrease, but its efficiency in terms of computational time improves when comparing to exact optimization methods. For problem instances 33–45, the CPLEX solution time exceeded 9,000 s, and CPLEX was able to identify a feasible solution but could not converge within the CPU time limit imposed. However, SOAPG obtained superior objective values with the solution time much less than 9,000 s. For subsequent large-scale problem instances, CPLEX could not provide a feasible solution within 10,000 s, while SOAPG could still provide good-quality solutions within reasonable computational time. Hence, according to the findings from the conducted experiments, the SOAPG algorithm has a high level of efficiency, which can be confirmed by the fact that the objective values of SOAPG are the optimal ones for small to medium-scale and small-scale problem instances. Furthermore, SOAPG can provide good solutions within an acceptable time range when solving large-scale problem instances.

Based on the results reported in Tables 17 and 18, the largest problem instances that could be solved by GA, SOA, and SA to optimality are problem instances 9, 10, and 9, respectively. SOAPG demonstrated a much more competitive performance and could obtain optimal solutions for 21 problem instances. Moreover, for all the considered problem instances, the objective values obtained by SOAPG are lower than those of GA, SOA, and SA, indicating the accuracy of SOAPG in solving the decision problem studied herein. For all the generated problem instances, the solving time of SOAPG was found to be slightly higher than that of GA, SOA, and SA. Such a pattern can be explained by the fact that during the SOAPG operations, in addition to global search, individual information exchange operations are also added. Nevertheless, the solving time of SOAPG is still within an acceptable time range. Therefore, according to the findings from the conducted experiments, the SOAPG algorithm is more prone to jumping out of local optimal solutions than traditional GA, SOA, and SA algorithms and demonstrates a high level of efficiency in terms of solution quality and computational time.

**Fig. 4:**
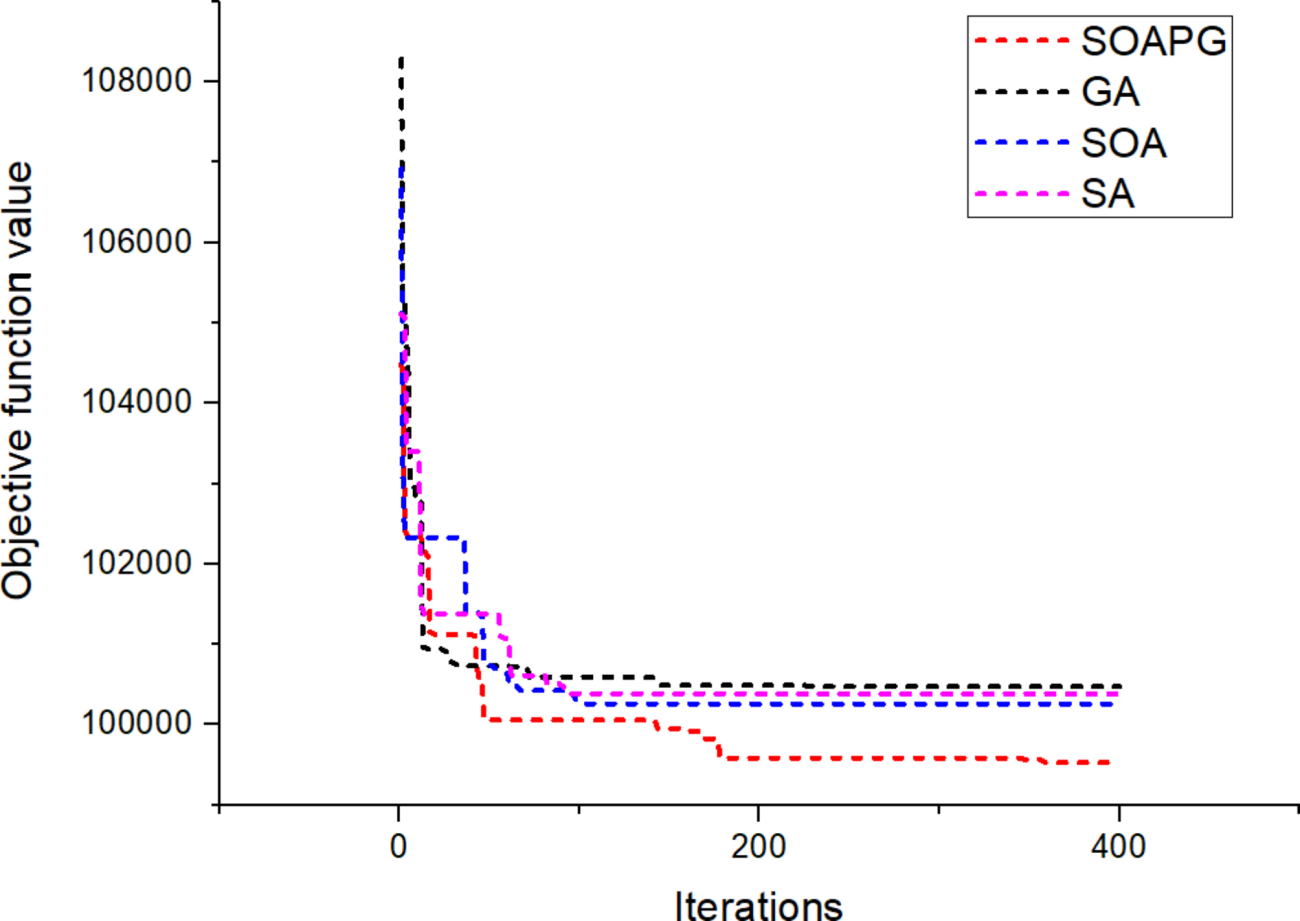
Convergence patterns of the four algorithms using problem instance 21 as an example.

To analyze the convergence speed and search capabilities of SOAPG, GA, SOA, and SA, the convergence patterns were evaluated for problem instance 21 for the four algorithms and are presented in Fig. 4. Similar tendencies in algorithmic performance were noticed for other problem instances as well. After 400 iterations, the objective values obtained are arranged in descending order as GA, SA, SOA, and SOAPG, respectively. From Fig. 4, it can be seen that under the same number of iterations, population size, and other parameters, SOA is more likely to jump out of the local optimal solutions during the search process when comparing to GA and SA, indicating the superiority of the SOA algorithm design. After adding crossover and mutation genetic operators, SOAPG shows advantages over the standard SOA in global and local search capabilities. Such a tendency can be explained by the fact that the new genetic operators can enhance communication between individuals in the population, making it easier to jump out of the local optimal solutions during the search process.

### Sensitivity analysis and managerial insights

The Guangzhou Port currently has four major port areas: Inner Port Area, Huangpu Port Area, Xinsha Port Area, and Nansha Port Area. The Inner Port of Guangzhou is composed of three port areas: Panyu, Wuhe, and Xintang. As such, six berthing bases are set up, including Huangpu Port Area, Xinsha Port Area, Nansha Port Area, Panyu Port Area, Wuhe Port Area, and Xintang Port Area. A total of 15 tugboats were considered during the sensitivity analysis, and the information for 25 tasks was regenerated using Tables 12, 13 and 14 as shown in Table 19. In addition, a maximum fuzzy delay time of (10,20,30,40) was set for all tasks.

**Table 19:** Information related to tasks.

Task	Tugboat demand	Requirements for horsepower	Number of tugs required for horsepower	Starting time (min)	Maximum waiting time (min)	Fuzzy service time
1	1	0	0	100	30	(10,13,15,20)
2	2	4000	2	45	30	(15,18,20,25)
3	2	3000	1	160	300	(15,18,20,25)
4	1	0	0	220	30	(10,13,15,20)
5	3	4000	2	280	30	(20,23,25,30)
6	1	0	0	340	30	(10,13,15,20)
7	2	4000	2	400	30	(15,18,20,25)
8	2	3000	1	460	30	(15,18,20,25)
9	2	0	0	510	30	(15,18,20,25)
10	3	5000	2	580	30	(20,23,25,30)
11	1	0	0	640	30	(10,13,15,20)
12	2	4000	2	700	30	(15,18,20,25)
13	2	3000	1	760	180	(15,18,20,25)
14	1	0	0	820	30	(10,13,15,20)
15	3	4000	2	880	30	(20,23,25,30)
16	1	0	0	940	30	(10,13,15,20)
17	2	4000	2	1000	30	(15,18,20,25)
18	2	3000	1	390	30	(15,18,20,25)
19	2	0	0	430	30	(15,18,20,25)
20	3	5000	2	320	30	(20,23,25,30)
21	1	0	0	1080	30	(10,13,15,20)
22	2	4000	2	910	30	(15,18,20,25)
23	2	3000	1	750	30	(15,18,20,25)
24	2	0	0	680	30	(15,18,20,25)
25	3	5000	2	600	30	(20,23,25,30)

In addition, the power of tugboats 1–15 (in HP) was set to 1600, 3000, 4000, 5000, 6000, 6800, 1600, 3000, 4000, 5000, 6000, 6900, 5000, 6000, and 6900, respectively. Before the start of the day’s operations, tugboats 2, 8, and 11 were assumed to be docked at the Huangpu Port Area berthing base; tugboats 5 and 14 were assumed to be docked at the Xinsha Port Area berthing base; tugboats 4, 9, and 13 were assumed to be docked at the Nansha Port Area berthing base; tugboats 6 and 15 were assumed to be docked at the Panyu Port Area berthing base; tugboats 1, 7, and 12 were assumed to be docked at the Wuhe Port Area berthing base; and tugboats 3 and 10 were assumed to be docked at the Xintang Port Area berthing base.

Although the optimal solution can be obtained for the 32nd problem instance, the efficiency of CPLEX significantly decreases when considering objective function 2 only, and the optimal solution can only be obtained for problem instances 1–6. Therefore, to ensure the rationality of the solution, this article applies both CPLEX and SOAPG algorithms to solve this problem instance. First, CPLEX was used to solve the optimization model with objective functions 1 and 3, then the SOAPG algorithm was used to solve the optimization model with objective function 2, and finally the SOAPG algorithm was used to solve the optimization model with all the objective functions considered and associated weights. When *N*=$\alpha$=$\beta$=$\lambda ^{\prime}$=0.5, solving the model with three objectives yielded the results presented in Table 20. Thus, the maximum value of objective 1 is 561,613.96, and the minimum value is 92,264.30; the maximum value of objective 2 is 5,323.22, and the minimum value is 0; and the maximum value of objective 3 is 1,125 and the minimum value is 1,095.

**Table 20:** Payoff matrices.

	f_1_(X)	f_2_(X)	f_t_(X)
*X* ^(1)^	92264.30	0	1125
*X* ^(2)^	561613.95	5323.22	1125
*X* ^(*t*)^	397634.53	0	1095

**Fig. 5:**
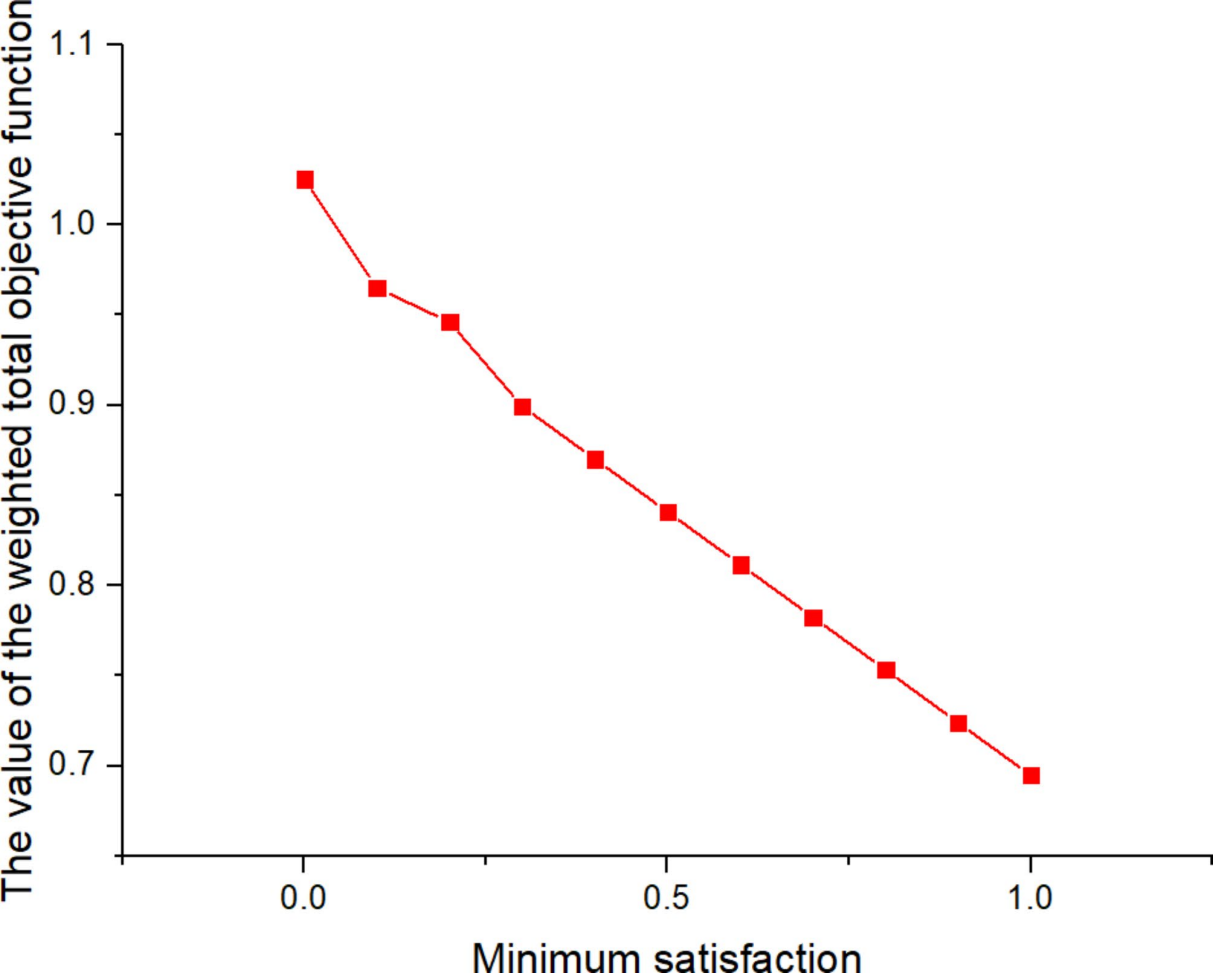
Behavior of the weighted total objective under the minimum satisfaction level change.

**Fig. 6:**
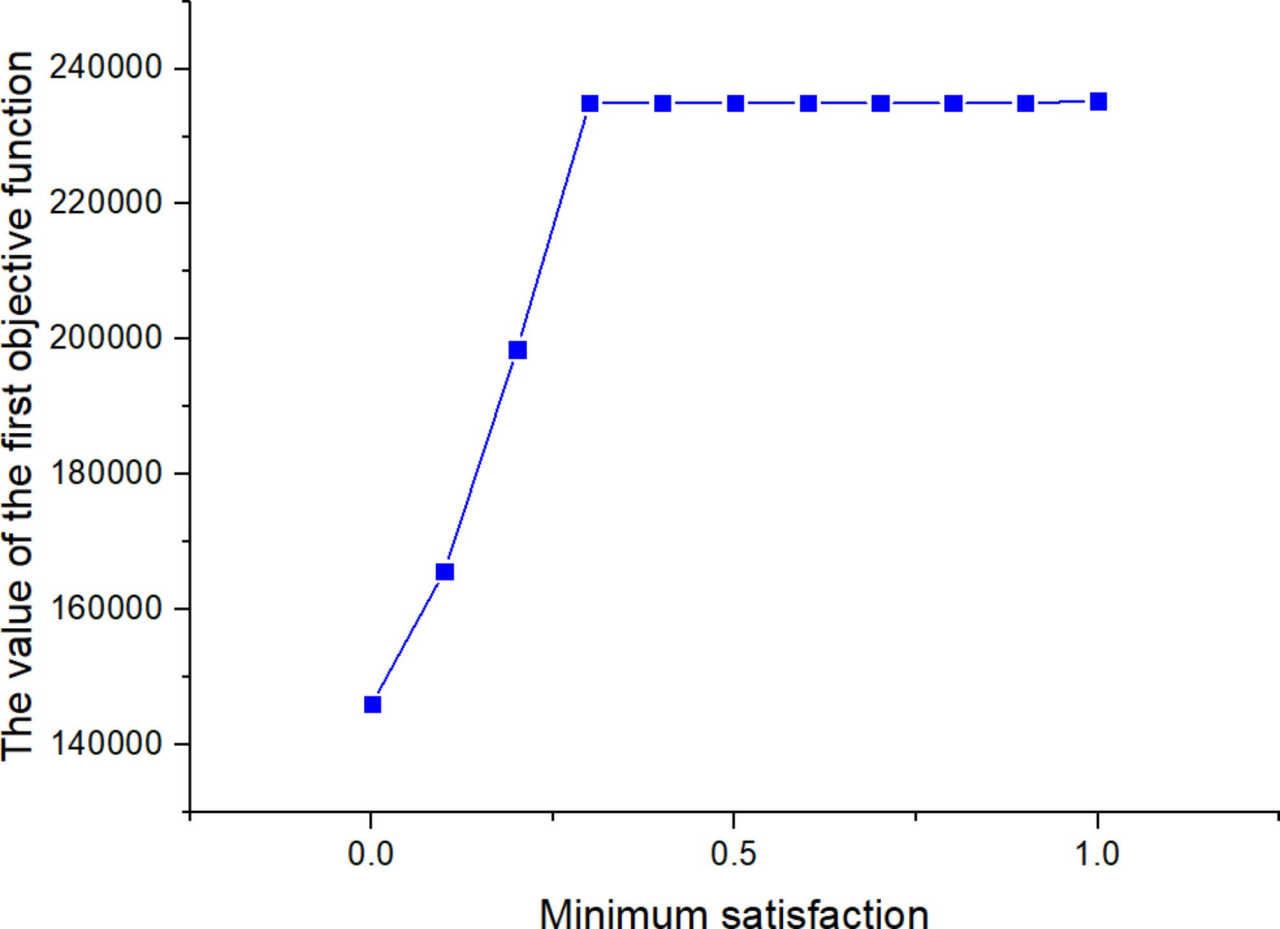
Behavior of objective 1 under the minimum satisfaction level change.

**Fig. 7:**
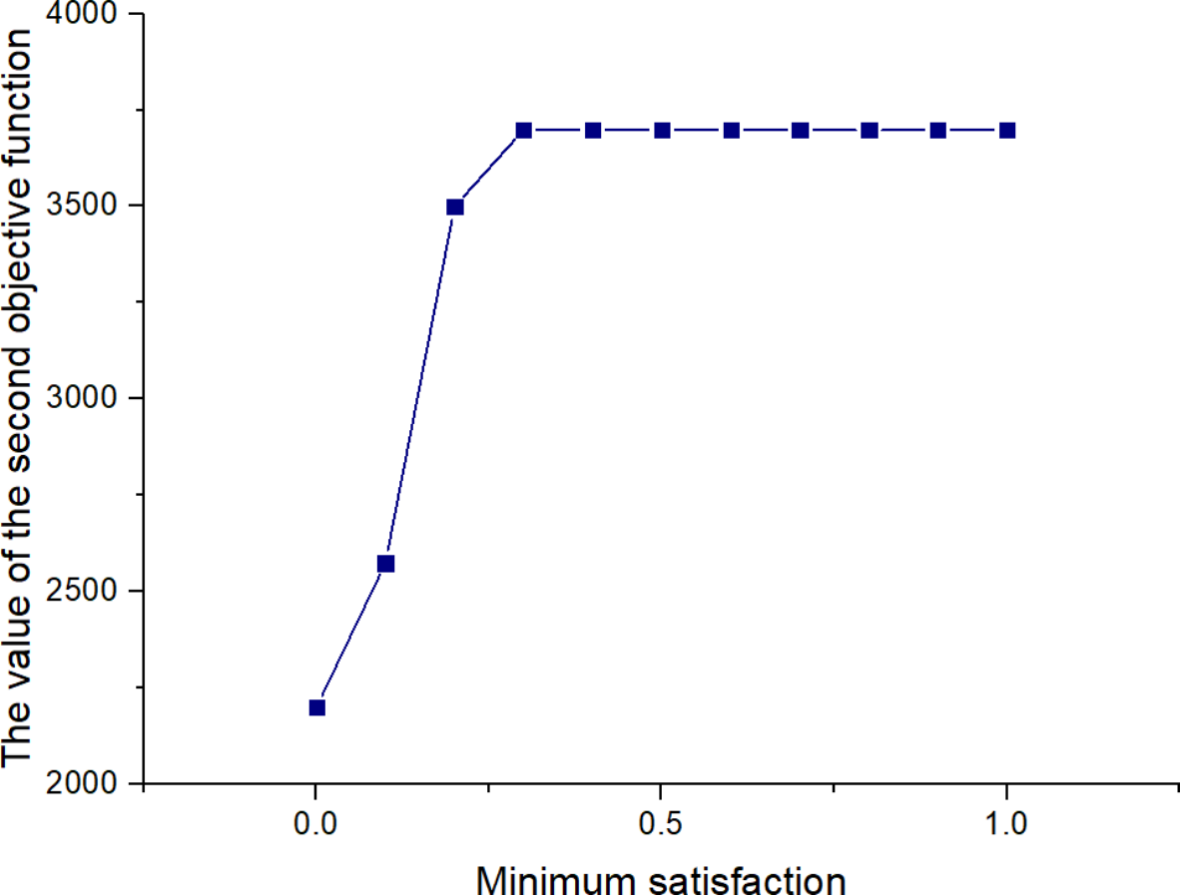
Behavior of objective 2 under the minimum satisfaction level change.

**Fig. 8:**
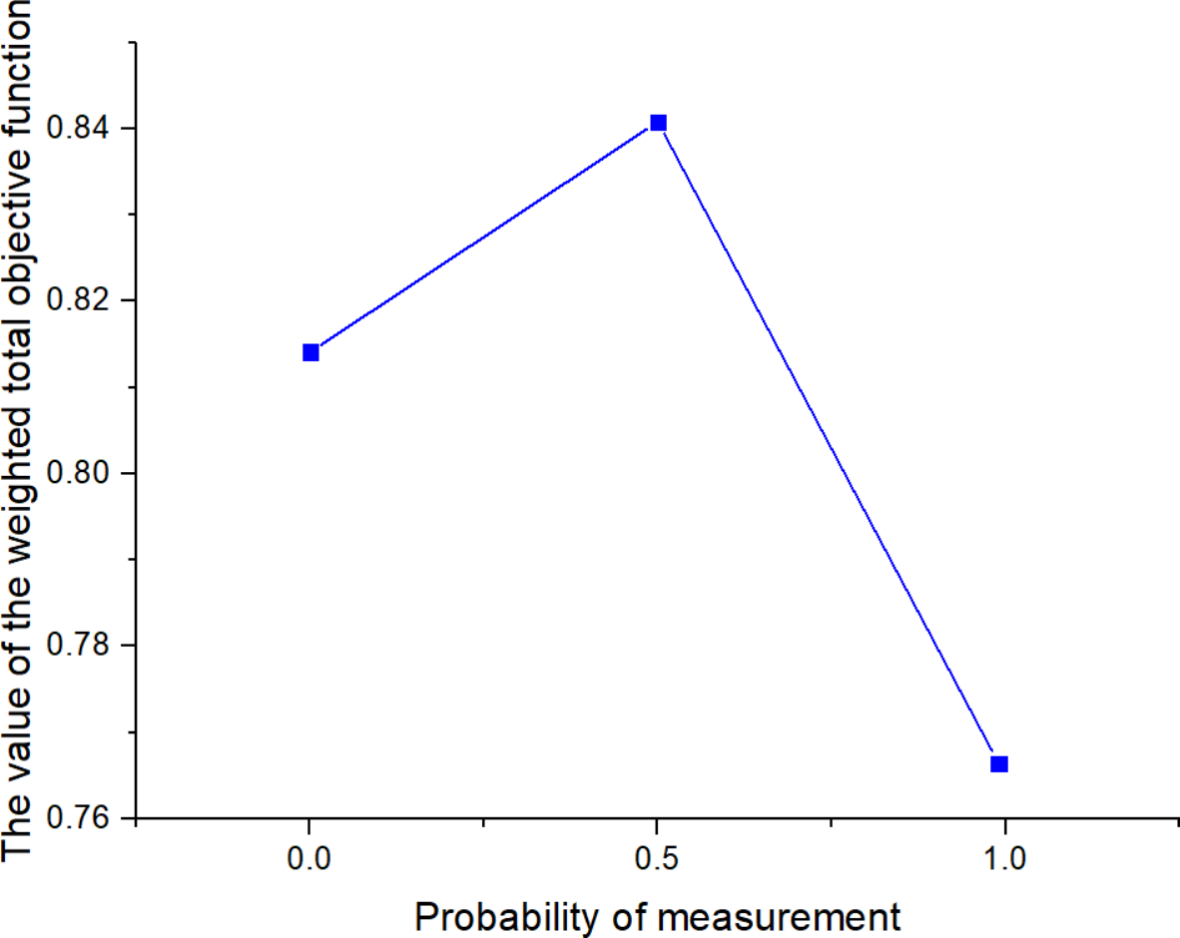
Behavior of the weighted total objective under the change of measure probability.

**Fig. 9:**
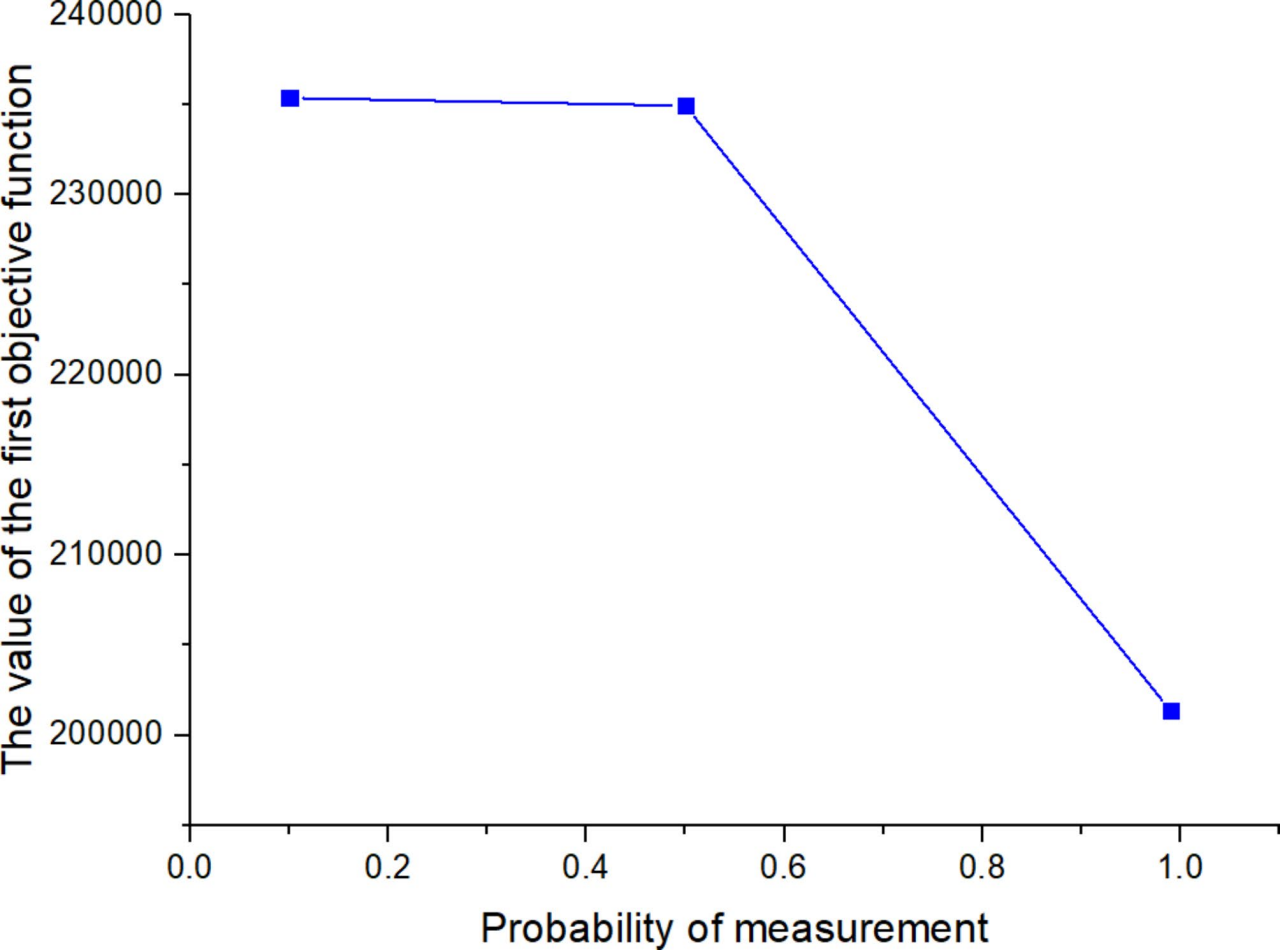
Behavior of objective 1 under the change of measure probability.

**Fig. 10:**
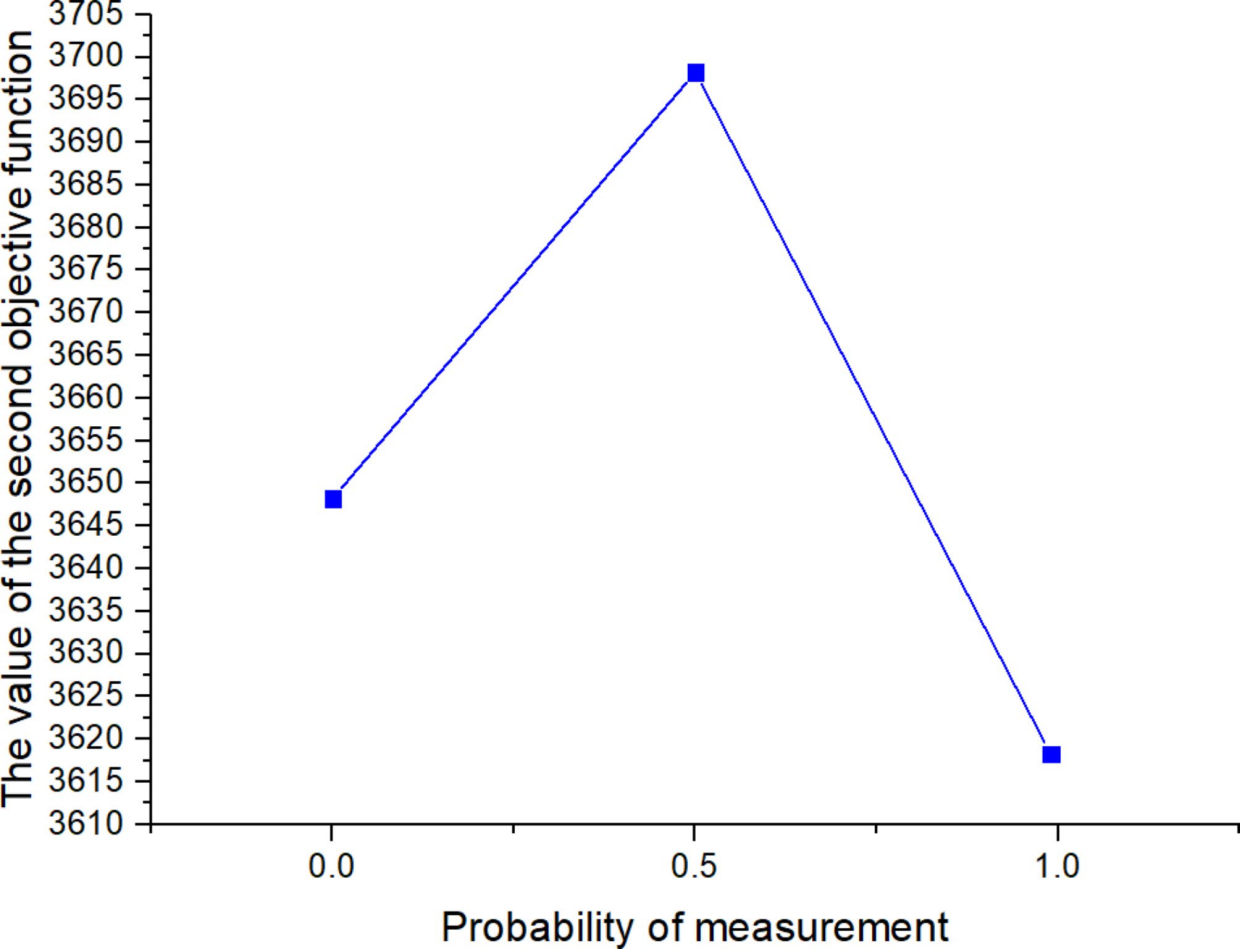
Behavior of objective 2 under the change of measure probability.

**Fig. 11:**
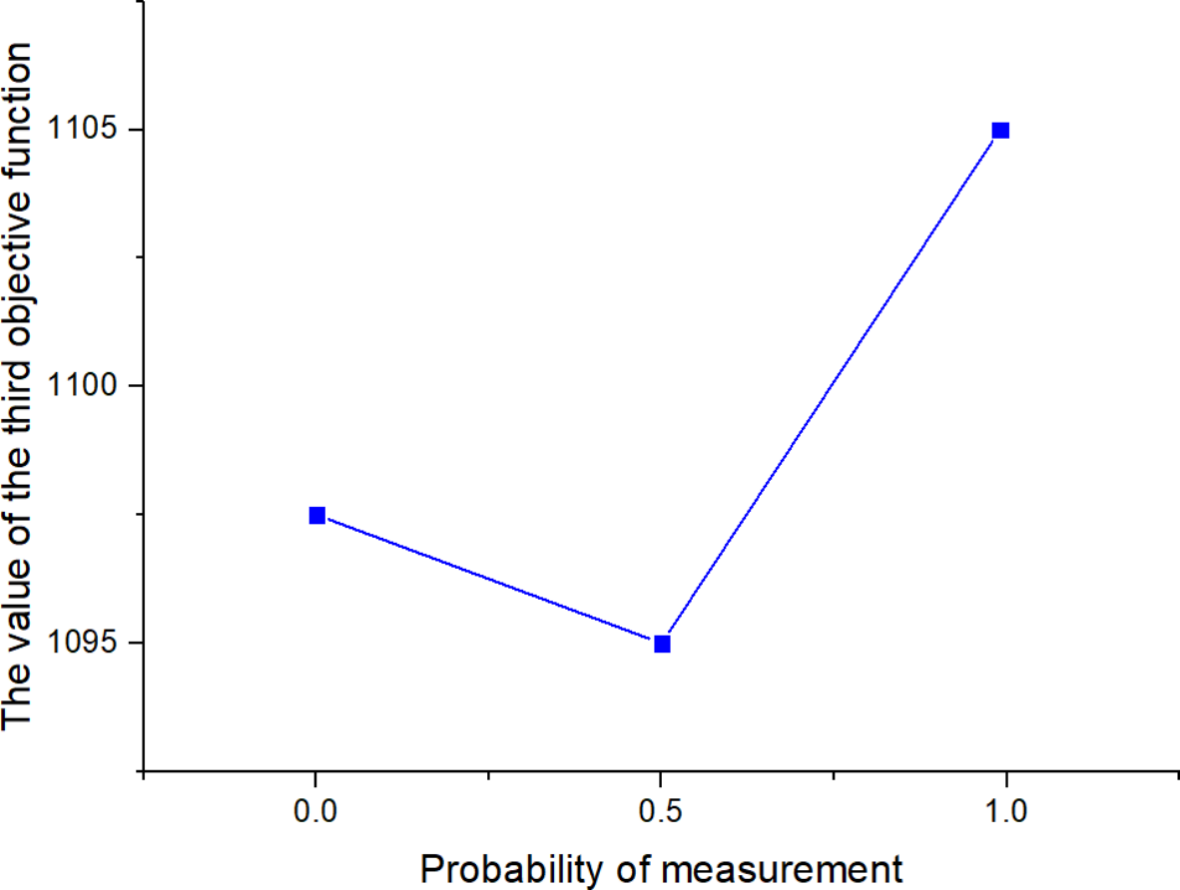
Behavior of objective 3 under the change of measure probability.

**Fig. 12:**
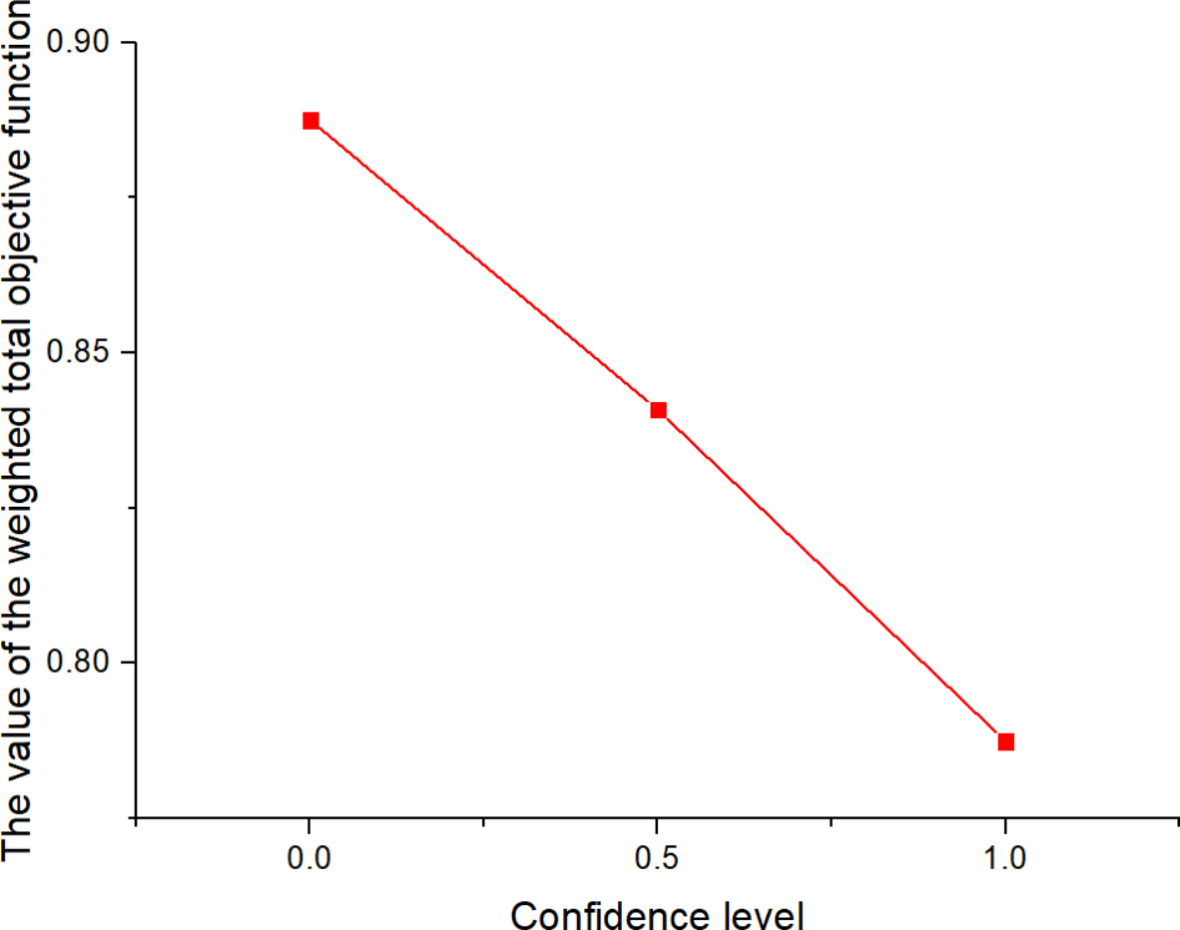
Behavior of the weighted total objective under the confidence level changes.

**Fig. 13:**
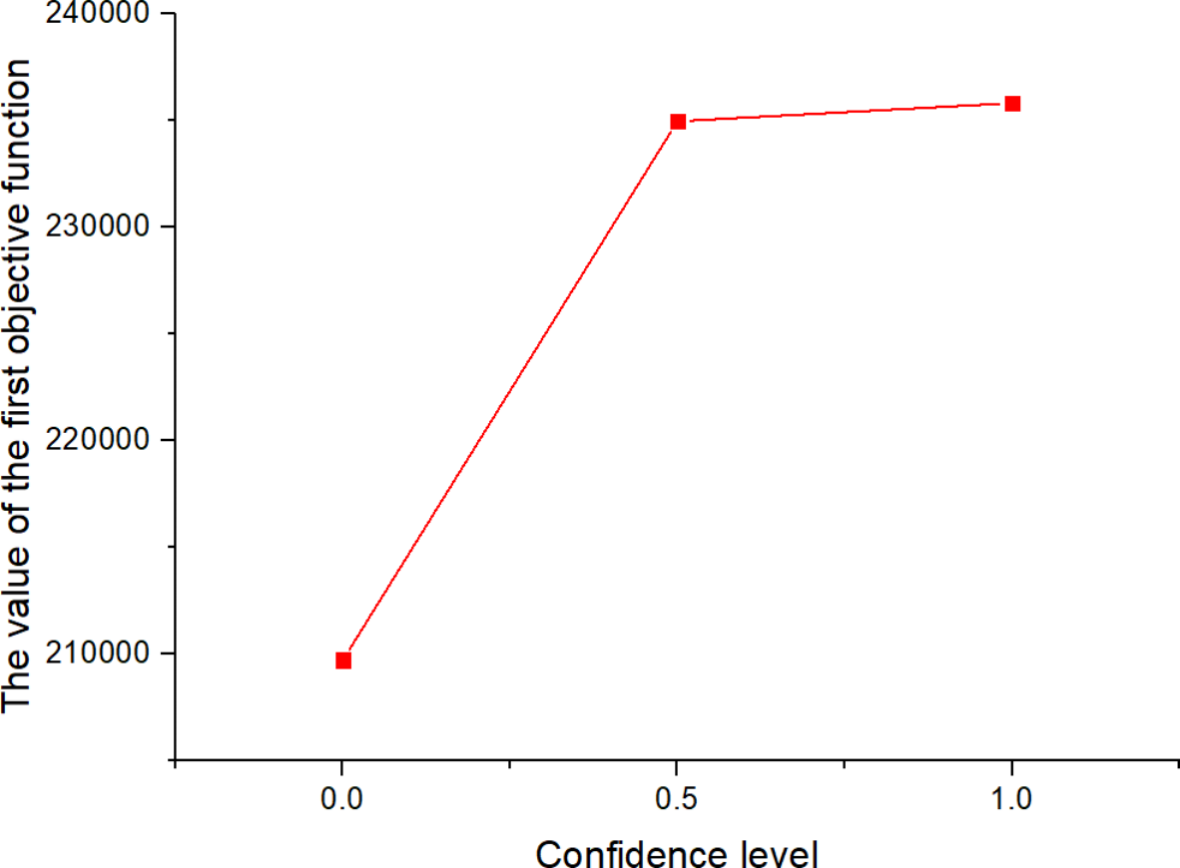
Behavior of objective 1 under the confidence level changes.

**Fig. 14:**
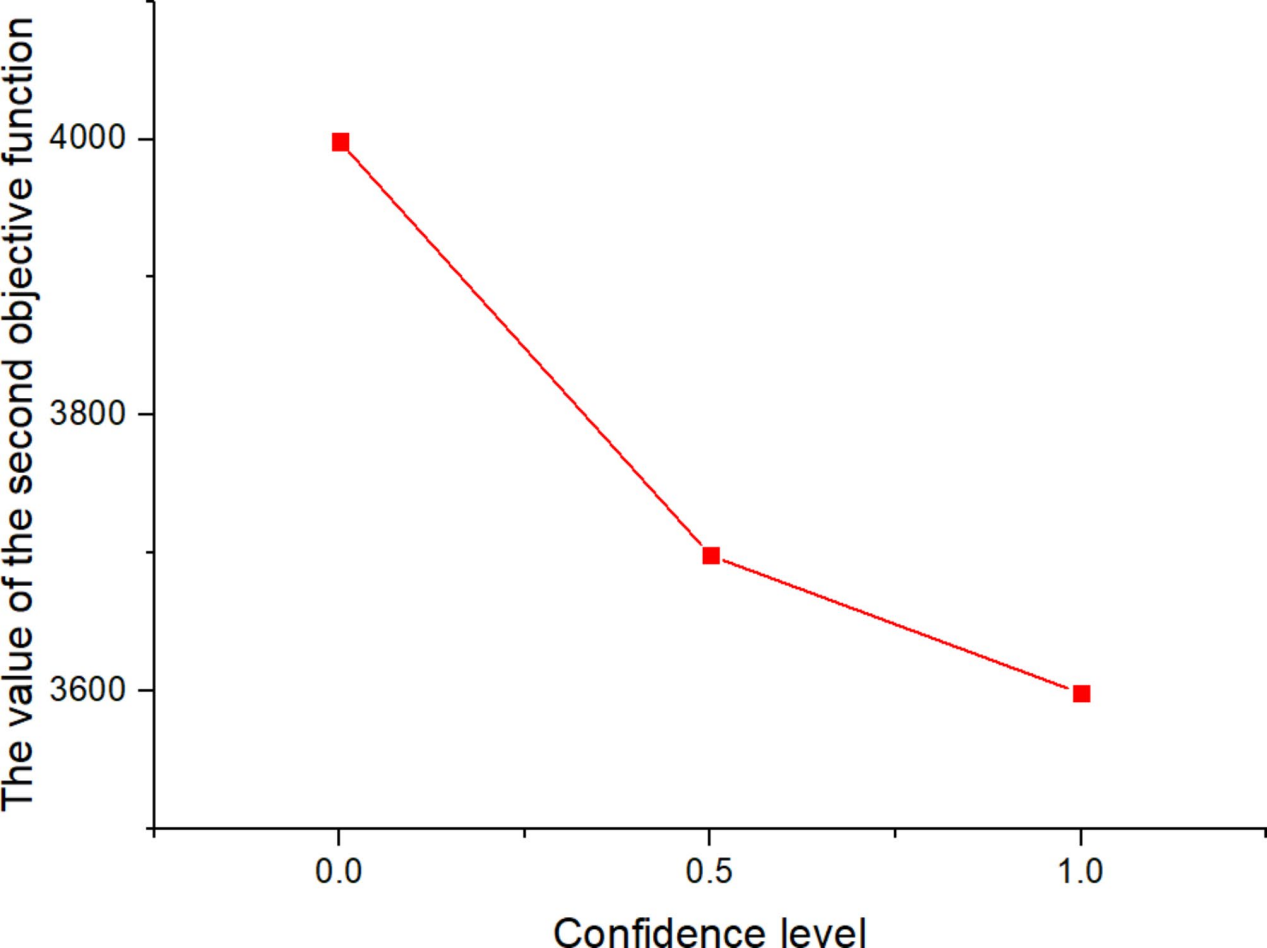
Behavior of objective 2 under the confidence level changes.

Next, the SOAPG algorithm was executed for various scenarios that were developed by changing the minimum satisfaction$\psi$level of the objectives, the decision maker’s preference estimates${\theta _1}$,${\theta _2}$and${\theta _3}$, the fairness coefficient *N*, and the values of fuzzy parameters$\alpha$,$\beta$and$\lambda ^{\prime}$. The recorded objective function values are presented in Table 21 for all the considered scenarios. Furthermore, Figs. 5, 6 and 7 were developed based on the data obtained for scenarios 12–16, 6, and 17–21; Figs. 8, 9, 10 and 11 were developed based on the data obtained for scenarios 26, 6, and 27; and Figs. 12, 13 and 14 were developed based on the data obtained for scenarios 28, 6, and 29.

**Table 21:** Changes in objective values for the considered scenarios of input parameters.

Scenario	$\:\psi\:$	$\:{\theta\:}_{1}$	$\:{\theta\:}_{2}$	$\:{\theta\:}_{3}$	*N*	$\:\alpha\:$	$\:\beta\:$	$\:{\lambda\:}^{{\prime\:}}$	Weighted total objective (aggregation function)	Objective 1	Objective 2	Objective 3
1	0.5	0.5	0.2	0.3	0	0.5	0.5	0.5	0.8395	236185.90	3698.22	1095
2	0.5	0.5	0.2	0.3	0.1	0.5	0.5	0.5	0.8408	234939.70	3698.22	1095
3	0.5	0.5	0.2	0.3	0.2	0.5	0.5	0.5	0.8408	234939.70	3698.22	1095
4	0.5	0.5	0.2	0.3	0.3	0.5	0.5	0.5	0.8408	234939.70	3698.22	1095
5	0.5	0.5	0.2	0.3	0.4	0.5	0.5	0.5	0.8408	234939.70	3698.22	1095
6	0.5	0.5	0.2	0.3	0.5	0.5	0.5	0.5	0.8408	234939.70	3698.22	1095
7	0.5	0.5	0.2	0.3	0.6	0.5	0.5	0.5	0.8408	234939.70	3698.22	1095
8	0.5	0.5	0.2	0.3	0.7	0.5	0.5	0.5	0.8408	234939.70	3698.22	1095
9	0.5	0.5	0.2	0.3	0.8	0.5	0.5	0.5	0.8408	234939.70	3698.22	1095
10	0.5	0.5	0.2	0.3	0.9	0.5	0.5	0.5	0.8408	234939.70	3698.22	1095
11	0.5	0.5	0.2	0.3	1.0	0.5	0.5	0.5	0.8408	234939.70	3698.22	1095
12	0	0.5	0.2	0.3	0.5	0.5	0.5	0.5	1.0254	145979.00	2199.01	1095
13	0.1	0.5	0.2	0.3	0.5	0.5	0.5	0.5	0.9650	165652.90	2573.81	1095
14	0.2	0.5	0.2	0.3	0.5	0.5	0.5	0.5	0.9461	198463.60	3498.63	1095
15	0.3	0.5	0.2	0.3	0.5	0.5	0.5	0.5	0.8993	234939.70	3698.22	1095
16	0.4	0.5	0.2	0.3	0.5	0.5	0.5	0.5	0.8701	234939.70	3698.22	1095
17	0.6	0.5	0.2	0.3	0.5	0.5	0.5	0.5	0.8116	234939.70	3698.22	1095
18	0.7	0.5	0.2	0.3	0.5	0.5	0.5	0.5	0.7824	234939.70	3698.22	1095
19	0.8	0.5	0.2	0.3	0.5	0.5	0.5	0.5	0.7532	234939.70	3698.22	1095
20	0.9	0.5	0.2	0.3	0.5	0.5	0.5	0.5	0.7239	234939.70	3698.22	1095
21	1	0.5	0.2	0.3	0.5	0.5	0.5	0.5	0.6947	235287.50	3698.22	1104.16
22	0.5	0.3	0.5	0.2	0	0.5	0.5	0.5	0.7560	249479.50	3698.22	1095
23	0.5	0.3	0.5	0.2	0.1	0.5	0.5	0.5	0.7754	234939.70	3698.22	1095
24	0.5	0.3	0.5	0.2	0.5	0.5	0.5	0.5	0.7754	234939.70	3698.22	1095
25	0.5	0.3	0.5	0.2	1	0.5	0.5	0.5	0.7754	234939.70	3698.22	1095.00
26	0.5	0.5	0.2	0.3	0.5	0.5	0.5	0	0.8141	235368.88	3648.22	1097.50
27	0.5	0.5	0.2	0.3	0.5	0.5	0.5	0.99	0.7664	201356.59	3618.22	1105
28	0.5	0.5	0.2	0.3	0.5	0	0	0.5	0.8875	209696.80	3998.32	1095
29	0.5	0.5	0.2	0.3	0.5	1	1	0.5	0.7874	235798.05	3598.22	1100

By observing the results obtained for scenarios 1–11 in Table 21, while keeping the coefficients other than the fairness coefficient constant, the weighted total objective changed from 0.8395 at the fairness coefficient of 0 to 0.8408 at the fairness coefficient of 0.1 and remained unchanged at 0.8408 thereafter. Objective 1 changed from 236,185.90 at a fairness coefficient of 0 to 234,939.70 at 0.1 and remained unchanged thereafter. Objective 2 and objective 3 remained unchanged at 3,698.22 and 1,095, respectively. This indicates that the change in the fairness coefficient has an insignificant impact on the fair allocation of tugboat task volume in the tugboat scheduling problem studied herein. Such a pattern can be justified by the fact that, in this example, there are a certain number of tugboats with the same horsepower that can meet the needs of 25 tasks. When the fairness coefficient changes, the tasks assigned to the original tugboat can be transferred to another tugboat with the same horsepower for operation, so the objective function value can be kept unchanged when the model solution changes. To illustrate the impact of coefficients other than the fairness coefficient, as shown in Table 21 for the data obtained for scenarios 22–25, when changing coefficients other than the fairness coefficient, the objective function trends appeared to be the same as those of recorded for scenarios 1–11.

By observing the data obtained for scenarios 12–16, 6, and 17–21 in Table 21; Figs. 5, 6 and 7, while keeping the coefficients other than the minimum satisfaction of the objective unchanged, the weighted total objective exhibited a linear decreasing trend during the change of the minimum satisfaction level of the objective from 0 to 1; objective 1 and objective 2 exhibited stable changes after increments from 0 to 0.3, respectively; and objective 3 remained stable during the change in the minimum satisfaction level from 0 to 0.9 and suddenly increased to 1,104.16 when it reached 1. This indicates that the change in the minimum satisfaction of the objectives had a significant impact on the tugboat scheduling problem studied herein. Decision makers of the tugboat company should choose a satisfactory tugboat plan suitable for port operations based on the actual conditions in the port.

By observing the data obtained for scenarios 26, 6, and 27 in Table 21; Figs. 8, 9, 10 and 11, while keeping the coefficients other than the measurement probability constant, the weighted total objective and objective 2 show an increase followed by a decrease after changing the measurement probability from 0 to 1; objective 1 shows a decreasing change; and objective 3 shows a decrease followed by an increase. This indicates that the impact of necessity measurement, possibility measurement, and feasibility measurement on the tugboat scheduling problem studied herein is obvious. Decision makers involved in the tugboat operations should fully consider the tugboat operational conditions after the vessel arrives at the port and choose a suitable measurement to develop a tugboat plan. According to the results in the provided example, the feasibility measure is the most suitable because it can maintain the maximum weighted total target satisfaction of the entire tugboat operations, lower fuel consumption, maximum total buffer time for dynamic tasks, and minimum latest end time.

By observing the data obtained for scenarios 28, 6, and 29 in Table 21; Figs. 12, 13 and 14, while keeping all the coefficients except the confidence level constant, the weighted total objective and objective 2 exhibited a quasi-linear decreasing change after the confidence level change from 0 to 1; objective 1 showed a stable and unchanged state after increasing; and objective 3 showed a steady and increasing state. This indicates that the change in the confidence level has a significant impact on the tugboat scheduling problem studied herein. Decision makers of the tugboat company should choose an appropriate confidence level based on the historical operational data of the tugboats used by the incoming vessels, as well as the hydrological and weather conditions at the port at that time, to ensure that the tugboat plan can be executed with low risk.

By comparing the solutions for objectives 1, 2, and 3, it was found that for the solutions for objectives 1 and 3, when tugboats serve the task, they can be allocated and scheduled according to the number of tugboat requirements${D_i}$for task *i*. However, for the solutions for objective 2, to maximize the total buffer time, the optimal solution for objective 2 assigns all the tugboats that meet the horsepower requirements to serve dynamic task 3. Although there is some irrationality in such a solution (referring to the allocation of extra tugboats to serve dynamic tasks), it indicates that if one wants to respond to the arrival of dynamic tasks and immediately dispatch tugboats for service, the decision makers of the tugboat company should keep all the tugboats that meet the horsepower requirements idle at the expected time of arrival of dynamic tasks to maintain the requirements of objective 2. From this perspective, the solutions satisfying objective 2 can be also viewed as reasonable.

The essence of the tugboat scheduling problem discussed in this study lies in the game-theoretic interaction between the tugboat scheduling of the tugboat company and the tugboat allocation of the port dispatcher. The Stackelberg game-theoretic method and multi-objective nature of the proposed model precisely solve the irrationality of objective 2, that is, adjusting the weights of the three objectives and the minimum satisfaction of the objectives to achieve a certain balance between the three goals of the tugboat company and the goal of port dispatcher in minimizing of the number of tugboats equipped for each task. As shown in the results for scenario 6 in Table 21, the weighted total objective satisfaction value is 0.8408, objective 1 is 234,939.70, objective 2 is 3,698.22, and objective 3 is 1,095.00. The analysis of the solution obtained for scenario 6 shows that 10 tugboat services were assigned for dynamic task 3, other tasks maintained the minimum tugboat demand, and all the coefficients had their intermediate values.

Due to the setting of the third task as a dynamic task in this section, it cannot represent generality. At the same time, when solving the 29 problem instances in this section, it took more than one hour to solve the model using CPLEX. To test the impact of dynamic tasks on the model and reduce the solving time, without changing the other input data (i.e., the coefficient values were the same as the values used in scenario 6 in Table 21), the following changes were made. Three objective functions were used as the objective functions of the M3 model, and then the 3rd, 8th, 13th, 18th, and 23rd tasks were set as dynamic tasks. The maximum waiting times for these five tasks were set to 300 min, 300 min, 180 min, 300 min, and 240 min, respectively. Finally, Eq. (58) was added as a supplementary constraint before executing CPLEX.

59 $$\sum\limits_{{k \in K}} {\sum\limits_{{l \in L}} {\sum\limits_{{i \in I}} {\left[ {\left( {{A_j}+{\tau _j}} \right){x_{klij}} - Y_{{klij}}^{1} - Y_{{klij}}^{2} - Y_{{klij}}^{3}} \right]} } } \leqslant NK \times {\tau _j},\quad \forall j \in I^{\prime}$$

The results show that the optimal solution for objective 1 could be obtained in 20 min (less time than the 21 problem instances described in Table 21), while 10 min were required for objectives 2 and 3 (see Table 22). The faster solution time can be explained by a tighter upper bound for objective function 2, reducing the search range and thus reducing the overall solution time required.

**Table 22:** Payoff matrices.

	f_1_(X)	f_2_(X)	f_t_(X)
*X* ^(1)^	91071.1	0	1125
*X* ^(2)^	1069068.32	19,800	1125
*X* ^(*t*)^	554516.03	0	1095

Because CPLEX takes more than two hours to solve the optimization model with the total objective combining objectives 1 through 3, the SOAPG algorithm can be viewed as the most practical solution approach. However, the introduction of Eq. (59) allowed achieving competitive computational time even for the cases with three objective functions considered at the same time.

## Conclusion

In recent years, an increase in China’s foreign trade cargo transportation has led to an increasing number of vessels docked at coastal ports, and ports are facing enormous operational pressure. As the main elements assisting vessels in entering and exiting ports, tugboats need to be scientifically managed. The current research proposed an optimization model for allocation and scheduling of tugboats. The developed model considered the impact of decisions of the tugboat operator and the port scheduling party on tugboat scheduling under the scenario of dynamic arrival of tasks and fuzzy tugboat operation time. The tugboat company aimed to minimize the total daily fuel consumption of tugboat operations, the total buffer time of dynamic tasks, and the total completion time as the objective functions, while the port dispatcher minimized the total number of tugboats allocated for the given tasks. The interactions between the tugboat operator and port dispatcher were modeled in the Stackelberg game-theoretic settings.

Based on the characteristics of the decision problem studied herein, a new priority encoding-based solution method was proposed and applied within the framework of the seagull optimization algorithm, which also incorporated genetic operators (i.e., the seagull optimization algorithm based on priority encoding and genetic operators – SOAPG). Finally, a comprehensive comparative analysis with the exact optimization method (CPLEX), genetic algorithm (GA), standard seagull optimization algorithm (SOA), and simulated annealing (SA) algorithm was conducted for a wide range of different problem instances to assess the effectiveness of the proposed algorithm. Numerous experimental results have shown that even for large-scale numerical examples, the proposed algorithm could provide optimal and high-quality solutions within acceptable computational time.

The results indicate that the proposed priority-encoding method was convenient and suitable for expressing the features of the problem studied in this research. In addition, the difference in CPU time required by SOAPG to solve the generated problem instances was not much higher than the time required by GA, SOA, and SA. In addition, compared to GA, SOA, and SA, the SOAPG algorithm achieved a smaller objective value of total fuel consumption in different situations. This indicates that the SOAPG algorithm was able to achieve better solution quality and robustness. Moreover, SOAPG demonstrated competitive performance in terms of solution quality and computational time when comparing to the exact optimization method (CPLEX). The applicability of the presented optimization model was also demonstrated through the sensitivity analysis, which was conducted by changing the fairness coefficient, uncertainty parameter correlation coefficients, and objective function correlation coefficients. The current research is of great significance for optimizing tugboat allocation and scheduling problems with uncertain situations and game-theoretic relationships. The results of this study are expected to provide scientific support for relevant port enterprises and stakeholders.

The current research can be further expanded in several aspects. In the future, we can fully consider how the tugboat companies and port dispatchers can collaborate in planning tugboat plans and develop more effective schedule alternatives. In addition, the encoding method proposed in this study can be considered universal and needs to be further deepened for its application in different maritime scenarios. Furthermore, better strategies need to be proposed to address the uncertainty of dynamic task arrivals, such as adopting a multi-scenario strategy, discretizing the period of dynamic arrival, studying the results brought by each arrival situation, and using certain robust methods to solve the resulting optimization model. Last but not least, the developed SOAPG algorithm can be compared to more advanced optimization algorithms, including customized heuristics, adaptive algorithms, island algorithms, diffused algorithms, and hyperheuristics.

## Electronic supplementary material

Below is the link to the electronic supplementary material.

Supplementary Material 1

## Data Availability

The datasets used and analyzed during the current study available from the corresponding author on reasonable request.
