# Supplementary material for: A multi-objective fuzzy programming model for port tugboat scheduling based on the Stackelberg game
Source: Sci Rep. 2024 Oct 23;14:25057. doi: 10.1038/s41598-024-76898-6 (PMC11499605; doi:10.1038/s41598-024-76898-6)
Supplement: Supplementary file 1 — Supplementary Material 1 [file 41598_2024_76898_MOESM1_ESM.docx]

The datasets used and analyzed during the current study available from the corresponding author on reasonable request.
